# Red/NIR-Emissive, Cadmium-Free Quantum Dots: Synthesis, Luminescence Mechanisms, and Applications

**DOI:** 10.3390/s26082473

**Published:** 2026-04-17

**Authors:** Yuna Son, Young Jun Kim, Dong Geun Han, Taesik Eom, Daeyoung Kim, Nahyeon Kim, Minsu Park

**Affiliations:** Department of Polymer Science and Engineering, Dankook University, 152 Jukjeon-ro, Yongin 16890, Gyeonggi-do, Republic of Koreayjkim99@dankook.ac.kr (Y.J.K.); 32202635@dankook.ac.kr (T.E.);

**Keywords:** cadmium-free quantum dots, red emission, PL mechanisms, QLEDs, NIR bioimaging

## Abstract

Red- and near-infrared (NIR)-emissive quantum dots (QDs) hold great promise in optoelectronic devices, sensors, and biomedicine owing to their advantages of low optical scattering, deep-tissue penetration, and compatibility with advanced photonic technologies. However, the toxicity of conventional cadmium (Cd)- and lead (Pb)-based QDs has led to growing demand for eco-friendly alternatives. Here, we provide a comprehensive review of sustainable classes of red/NIR-emissive QDs, including indium phosphide (InP), I-III-VI chalcogenides (CuInS_2_, AgInSe, and so on), group-IV (Si, Ge, and SiGe) nanocrystals, and carbon-based QDs (graphene QDs or carbon dots). InP QDs are leading candidates for display technologies due to their high efficiencies and narrow bandwidths in emission properties, enabled by advanced core/shell engineering. In contrast, I-III-VI chalcogenides, group-IV, and carbon-based QDs offer advantages for biocompatible NIR bioimaging, photothermal therapy, and silicon photonics integration. We discuss synthesis strategies for achieving long-wavelength emission, the mechanisms of red/NIR photoluminescence (PL), and representative applications in displays, sensors, and bioimaging. Finally, we outline the remaining challenges, such as large-scale manufacturing and long-term stability, which should be addressed for commercial and clinical viability.

## 1. Introduction

Quantum dots (QDs) are typically defined as inorganic, semiconductor nanocrystals that possess various advantages, such as high photoluminescence quantum yields (PLQYs), a narrow full width at half maximum (FWHM), and tunable bandgaps [1,2,3]. The size-dependent photoluminescence (PL) of the QDs arises from quantum confinement effects, which occur when the spatial extent of charge carriers is confined to dimensions smaller than the exciton Bohr radius (*a*_B_), leading to discrete energy levels. In particular, QDs emitting in the red- to near-infrared (NIR) region (610–1700 nm) provide significant benefits for both photonic and biological applications [4,5,6,7]. In display technologies, deep-red emission (630–670 nm) is advantageous for achieving enhanced color gamut and saturation [8,9,10]. In specialized lighting applications, such as horticulture, emission in the red spectral range overlapping with chlorophyll absorption bands (650–750 nm) promotes photosynthetic efficiency and plant growth. In biological applications, deep-red and NIR emission (690–1700 nm) falls within the biological transparency windows (NIR-I: 700–950 nm; NIR-II: 1000–1700 nm) [11]. Hereby, absorption and scattering by endogenous chromophores, such as hemoglobin and water, are significantly reduced [12], which enables deeper tissue penetration (up to ~14 mm in ex vivo studies) and a higher signal-to-noise ratio, facilitating high-resolution deep-tissue imaging.

Over the past decades, cadmium selenide (CdSe)-based II–VI QDs have achieved the highest level of commercial maturity, owing to their technological advantages, scalable manufacturing, and superior performance. However, their continued reliance is increasingly restricted by environmental regulations (e.g., RoHS) due to the intrinsic toxicity of Cd, motivating the development of non-cadmium material systems with diverse physicochemical and luminescent properties. Inorganic material-based alternatives may include III-V semiconductors (e.g., indium phosphide, and InP) [13,14,15,16], I-III-VI compounds (e.g., CuInS_2_ (CIS) and AgInSe_2_ (AIS)) [17,18,19,20], and group-IV materials (e.g., Si and Ge) [21,22,23,24]. Organic alternatives may comprise carbon-based nanomaterials, such as carbon dots (CDs) [25,26,27,28,29,30,31] and graphene quantum dots (GQDs) [32,33,34,35,36,37,38,39].

InP is a direct semiconductor that exhibits high emission tunability over the visible region [40]. A key to achieving highly efficient, red-emissive InP QDs is the precise construction of core/multi-shell heterostructures, for example, InP/ZnSe/ZnS. Meanwhile, the luminescence mechanism in I-III-VI chalcogenide QDs (e.g., CIS and AIS) is predominantly governed by defect-mediated radiative recombination [41]. For example, the PL emission of CIS QDs originates from donor–acceptor pair (DAP) transitions associated with intrinsic point defects, such as copper vacancies (*V*_Cu_). Tuning the density and energy distribution of these DAP states enables emission color modulation from green to red, primarily through stoichiometric controls (e.g., Cu/In ratio). The Si and Ge QDs are also attractive due to their low toxicity and compatibility with silicon-based processing. However, their bulk forms possess indirect bandgaps, resulting in intrinsically low PLQYs. Efficient emission is achieved under quantum confinement. Si QDs show size-dependent visible emission (e.g., ~680 nm for ~3.1 nm particles), with PLQYs up to ~90% [42,43]. Ge QDs, with a narrower bulk bandgap (0.66 eV), are intrinsically suited for NIR emission, making them promising for silicon photonics and deep-tissue bioimaging applications [44,45].

GQDs/CDs offer distinct advantages over the inorganic QDs described above, such as excellent water solubility [46,47], unique luminescence mechanisms, and ultra-low cytotoxicity. Efforts to realize the unique photonic functionality in graphene stemmed from attempts to overcome its inherent zero-bandgap electronic structure. This led to the discovery of PL emission in nanoscale fragments of the graphene, termed GQDs. The emission properties of the GQDs were initially associated with the quantum confinement effect [48,49]. However, subsequent research has revealed more complex luminescence mechanisms, primarily originating from two co-existing modes: (i) intrinsic emission from small, confined sp^2^ carbon subdomains, and (ii) extrinsic emission induced from edge state and surface defects, often involving oxygen-containing functional groups [50,51]. A critical challenge is establishing a clear understanding of this surface chemistry, as suppressing non-radiative extrinsic pathways is essential for achieving highly efficient intrinsic emission [52]. In particular, achieving efficient red emission in GQDs and CDs requires targeted bandgap engineering strategies [53], which will be discussed in the subsequent section.

**Scheme 1:**
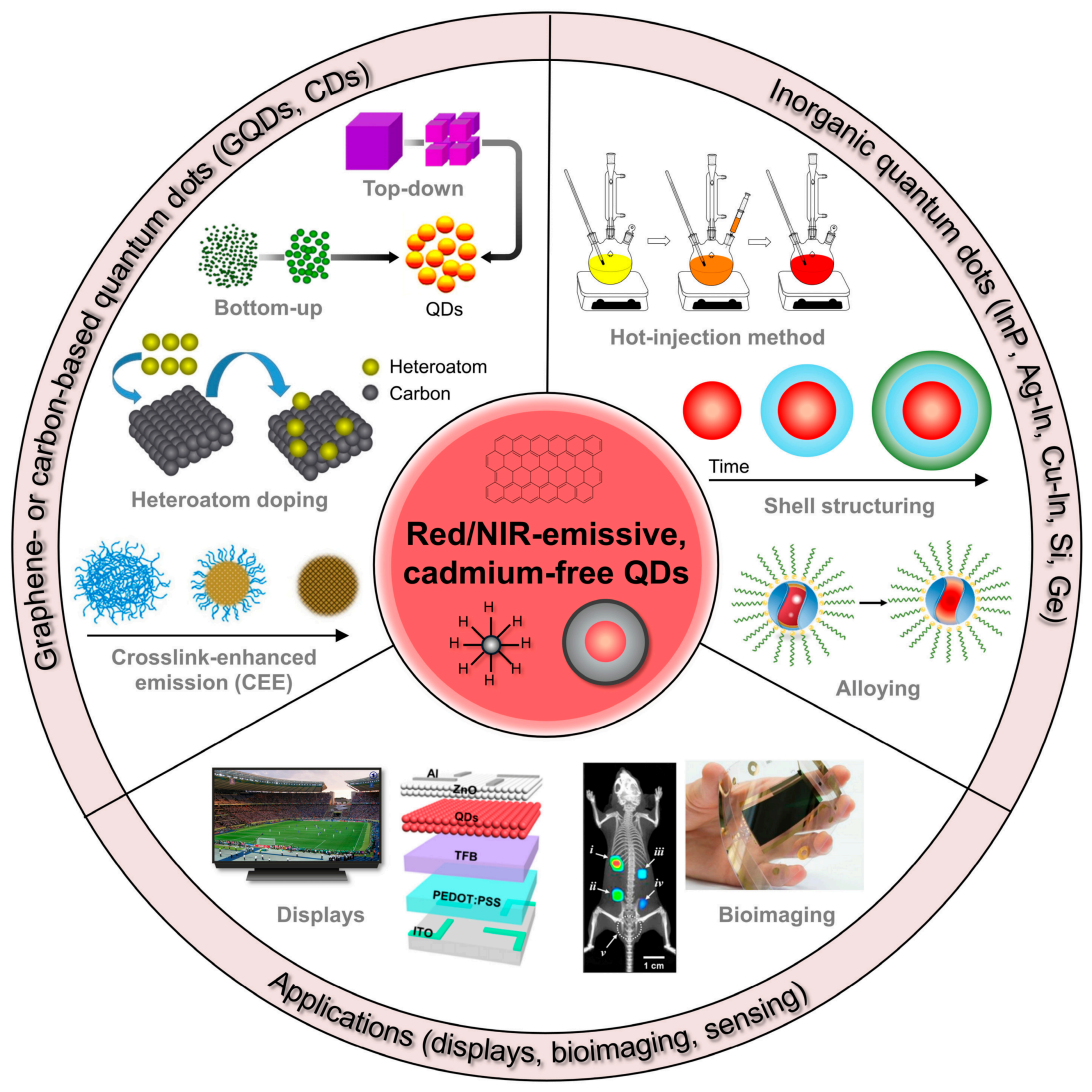
Outline of this review. Reproduced from [54,55,56,57], with permission from Wiley–VCH. Copyright (2021, 2022), the Royal Society of Chemistry. Copyright (2021), under Creative Commons Attribution 4.0 (CC BY). Copyright (2022). Reproduced from [58] with permission from the American Chemical Society, under CC-BY-NC-ND 4.0. Copyright (2025).

This review provides a comprehensive overview of the synthesis, luminescence mechanisms, and applications of red-emissive QDs based on several types of Cd-free materials (Scheme 1). While it focuses mainly on emission spanning the red to NIR-I region (610–950 nm), the characteristics of the NIR-II region (1000–1700 nm) can be selectively addressed in sensor applications, such as time-gated detection. It first raises environmental and regulatory limitations regarding existing commercial Cd-based QDs and suggests that several types of non-toxic inorganic/organic QD systems can serve as alternatives, each leveraging their own unique material strategies. Section 2 outlines the synthetic methodologies specifically designed to achieve red/NIR emission, including hot-injection methods for InP, AIS, and CIS QDs; plasma- or non-plasma-based synthesis for Si QDs; epitaxial growth or colloidal synthesis of Ge QDs; and various top-down and bottom-up approaches for GQDs and CDs. In Section 3, representative strategies for realizing highly efficient red/NIR emissions from each material are presented with a comparative discussion. Section 4 systematically evaluates the performance and technological maturity of these materials in three key application sectors: QD light-emitting diodes (QLEDs), sensors, and advanced biomedical imaging. Lastly, we will discuss the remaining challenges and the outlook for red/NIR-emissive, Cd-free QDs, aiming to guide a comprehensive perspective of this research field.

## 2. Synthesis of Red/NIR-Emissive QDs

### 2.1. Carbon- or Graphene-Based QDs

**Figure 1:**
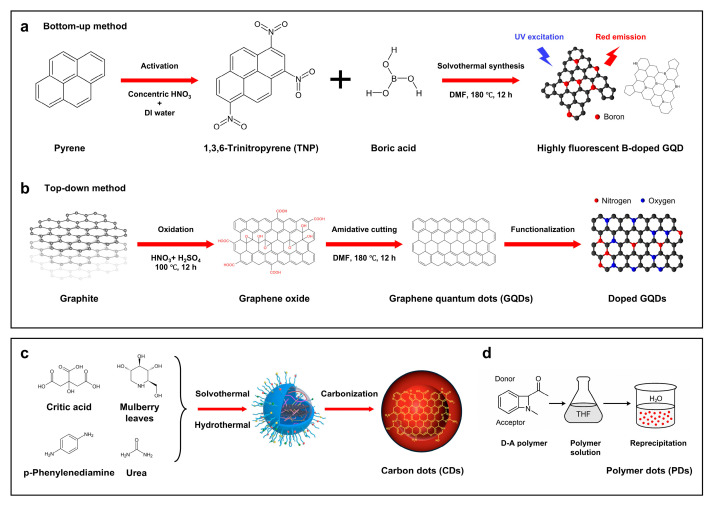
Synthetic strategies for graphene quantum dots (GQDs) or carbon dots (CDs). (**a**) Bottom-up synthesis of red-emissive GQDs (R-GQDs): solvothermal treatment of pyrene and boric acid. (**b**) Top-down synthesis of R-GQDs: modified Hummer’s method, followed by functionalization. (**c**) Bottom-up synthesis of red-emissive CDs (R-CDs): solvo- and hydrothermal treatment, followed by carbonization. (**d**) Synthesis of red-emissive polymer dots (R-PDs) via donor–acceptor (D–A) molecular design, followed by reprecipitation. Reproduced from [61] with permission from Elsevier. Copyright (2021).

Figure 1 summarizes representative synthetic strategies for red-emissive GQDs and CDs. Figure 1a illustrates a bottom-up synthesis for GQDs through a solvothermal process, using pyrene and boric acid. First, the pyrene is converted to 1,3,6-trinitropyrene (TNP) in concentric nitric acid and deionized water. Subsequent solvothermal reaction with boric acid in dimethylformamide (DMF) produces highly fluorescent boron (B)-doped GQDs. It exhibits red emission centered at 617 nm, with a FWHM of 31.7 nm under ultraviolet (UV) excitation [59]. Figure 1b depicts a typical top-down approach, using graphite as a sp^2^ carbon precursor. The graphite is exfoliated by acidic oxidation using a nitric acid/sulfuric acid mixture to yield graphene oxide (GO), followed by amidative cutting in DMF to produce nanoscale, low-oxidation GQDs. Functionalization of GQDs with aniline derivatives enables red emission with a major peak at 605 nm [60]. Figure 1c presents a bottom-up synthesis for CDs, where representative molecular precursors, such as citric acid, mulberry leaf extract, *p*-phenylenediamine, and urea, undergo solvothermal or hydrothermal treatment. During the process, red-emissive CDs that contain mixed sp^2^/sp^3^ carbon domains are generated through carbonization [25,26,27,61,62,63,64,65,66]. Another class of carbon-based QDs indicates polymer dots (PDs), which are synthesized via reprecipitation of donor–acceptor polymers dissolved in tetrahydrofuran (THF) (Figure 1d). The resulting PDs exhibit deep-red emission centered at 660 nm [67].

### 2.2. Inorganic Core–Shell QDs

#### 2.2.1. Indium-Containing Core-Based QDs

**Figure 2:**
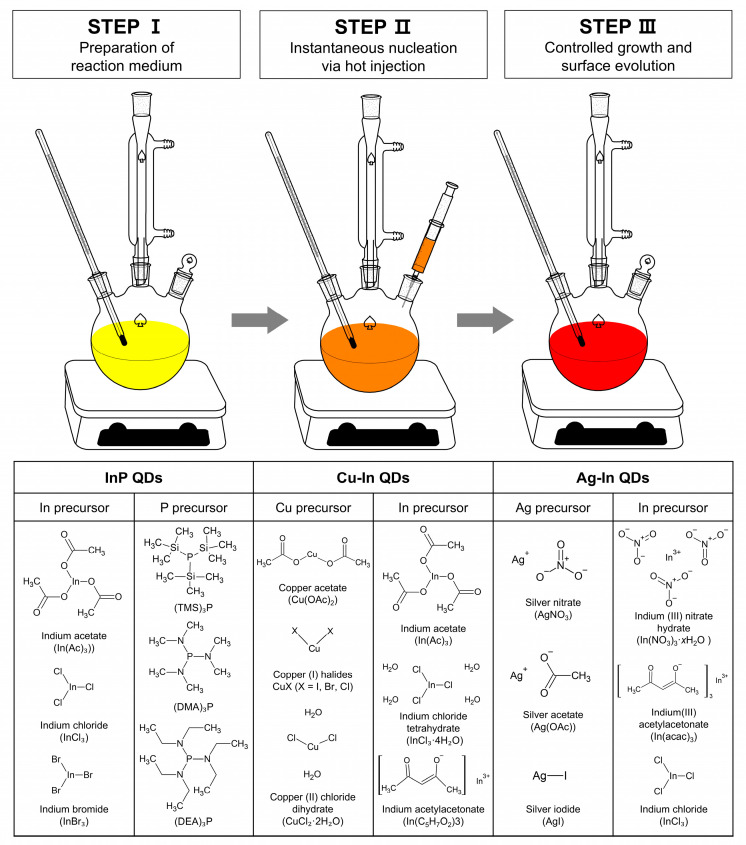
A schematic illustration of the hot-injection method and representative metal precursors used for the synthesis of indium phosphide (InP), copper–indium (Cu-In) core-based, and silver-indium (Ag-In) core-based QDs.

Indium-containing core-based QDs, such as InP, CIS, and AIS QD systems, require precise synthetic control to achieve compositional uniformity and efficient core/multi-shell heterostructures. Among various approaches, the hot-injection method serves as a representative route for the materials mentioned above. It enables an accurate separation of the nucleation and growth stages, which is essential for producing uniform particles. It also allows for the use of diverse organic capping ligands, facilitating monodispersity and high crystallinity. Figure 2 shows a schematic illustration of the hot-injection method and representative molecular precursors used for the synthesis of InP, AIS, and CIS core-based QDs. Step I indicates preparation of the reaction medium through heating of metal precursors in coordinating solvents with a high boiling point. Examples of metal precursors include indium carboxylates or halides, copper acetate, and silver nitrate, which can be used individually or in a combined form, depending on the core system to be formed. Step II represents a sudden supersaturation and subsequent instantaneous nucleation. This involves a rapid injection of a reactive anion precursor into the hot reaction medium. In InP cores, for example, tris(trimethylsilyl)phosphine ((TMS)_3_P) is commonly used as a phosphorous precursor. To form ternary alloyed cores (e.g., CIS or AIS), chalcogen precursors (e.g., dodecanethiol or selenium powder) can be used. Finally (Step III), the regulation of temperature and reaction time enables precise control of QD size and chemical composition via a controlled growth and surface evolution (La Mer). Many other synthetic approaches have been explored for target material systems [8,13,16,68,69,70].

Various indium precursors have been employed in the synthesis of InP QDs. Representative examples include halide-based compounds, such as InCl_3_, InBr_3_, and InI_3_, as well as carboxylate-based compounds like indium acetate (In(OAc)_3_) and indium palmitate (In(PA)_3_) [8,16,68]. These precursors are selected based on their reactivity, solubility, and interaction with ligands, which critically influence nucleation kinetics and growth control during synthesis. The most commonly used phosphorus precursor is (TMS)_3_P due to its high reactivity, which enables rapid nucleation and precise size control. Combined use of In(OAc)_3_ and (TMS)_3_P is widely recognized as an optimal pair for synthesizing highly luminescent InP/ZnSe/ZnS QDs, yielding narrow emission band-widths (FWHM ≈ 35 nm) and high PLQYs up to 95% [16,71,72,73]. However, a rapid consumption of (TMS)_3_P during the initial nucleation often requires additional injection of phosphorus precursor [8,16]. As a cost-effective alternative, tris(dimethylamino)phosphine (P(N(CH_3_)_2_)_3_) is suitable for facile solvothermal synthesis and low-temperature aminophosphine-based syntheses, but it often yields InP QDs with lower PLQYs and broader emission bandwidths [13,16]. Tris(dimethylamino)phosphine ((DMA)_3_P) has also been investigated as a phosphorus precursor due to its lower reactivity, enabling better QD size control during growth [8]. The choice of zinc source significantly affects shell crystallinity, lattice compatibility, and optical properties of the resultant QDs. For shell formation, zinc chloride (ZnCl_2_), zinc oleate (ZnOA), and zinc acetate (Zn(OAc)_2_, ZnAc_2_) are commonly used [13,16,74]. While ZnCl_2_ contributes to suppressing surface defects and achieving homogeneous ZnS shell formation, the introduction of a ZnSe buffer layer prior to ZnS deposition alleviates lattice mismatch and enhances spectral purity. ZnOA promotes stable colloidal synthesis as it interacts well with organic ligands. To supply chalcogen elements during shell formation, trioctylphosphine sulfide (TOP-S) and trioctylphosphine selenide (TOP-Se) are widely used [13]. These reagents facilitate controlled shell deposition in organic solvents and largely affect the thickness, crystallinity, and surface properties of the final core/shell nanostructures. Collectively, these results underscore that rational precursor design, combined with optimized shell engineering, is key to achieving high-quality red-emissive InP, AIS, and CIS core-based QDs [13,15,16,69,71,72,73,74].

#### 2.2.2. Silicon (Si) QDs

**Figure 3:**
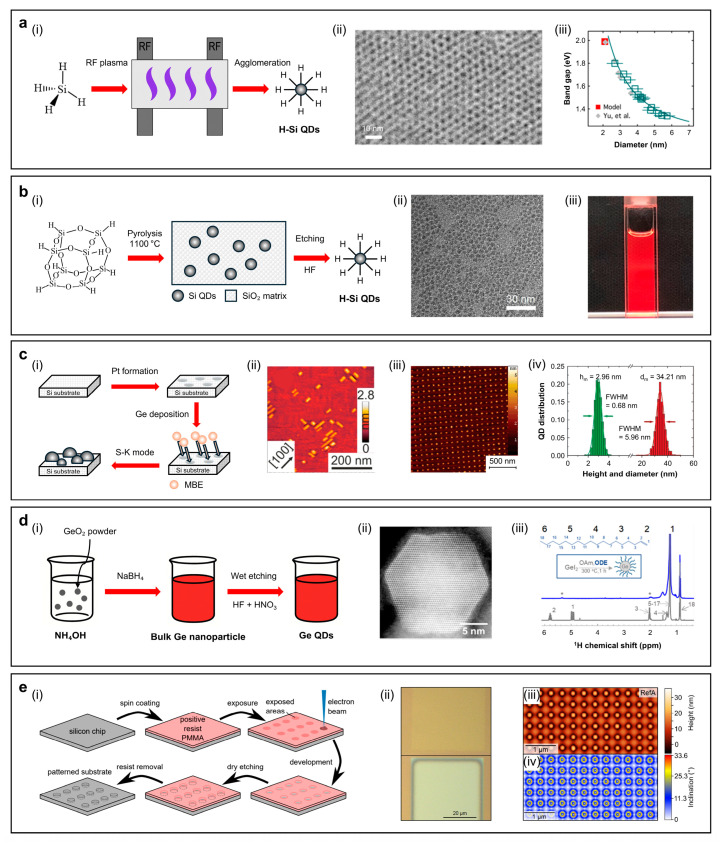
(**a**,**b**) A schematic illustration of two representative synthetic strategies for silicon quantum dots (Si QDs). (**a**) The radio-frequency (RF) plasma-based method (**i**), the TEM image of Si QDs (**ii**), and bandgap versus diameter of Si QDs (**iii**); reproduced from [76], with permission from the American Chemical Society. Copyright (2020). (**b**) Non-plasma-based pyrolysis, using a hydrogen silsesquioxane (HSQ) (**i**), TEM image (**ii**), and a digital image (**iii**) of decyl-terminated Si QDs prepared by thermal hydrosilylation; reproduced from [78] with permission from the American Chemical Society. Copyright (2022). (**c**,**d**) A schematic illustration of two representative synthetic strategies for germanium quantum dots (Ge QDs): (**c**) epitaxial growth via molecular beam epitaxy (MBE) (**i**), an 2D atomic force microscopy (AFM) image of Ge QDs (**ii**), an AFM image of 10 period stacks of Ge islands and Si spacer layers (10 nm) deposited on a prepatterned area (**iii**), and the corresponding histogram illustrating the distribution of the size and the height of the Ge QDs in (**iv**); reproduced from [23], with permission from the American Chemical Society under Creative Commons (CC BY). Copyright (2016). Reproduced from [86], with permission from the American Chemical Society. Copyright (2007). (**d**) The colloidal synthesis of Ge QDs (**i**), annular dark-field scanning transmission electron microscopy (ADF-STEM) images of octadecene (ODE)-capped Ge QDs (**ii**), and ^1^H NMR spectra of Ge QDs, synthesized in oleylamine (blue line) and ODE in chloroform-*d* (grey line) (**iii**). Reproduced from [24], with permission from the American Chemical Society. Copyright (2025). (**e**) An illustration of the pit-patterning process (**i**), an optical image of successfully exposed (**top**) and overexposed (**bottom**) pit-patterned areas (**ii**), an AFM image (**iii**), and inclination maps (**iv**) showing the facets of the dome-shaped dots. Reproduced from [91], with permission from Springer Nature under Creative Commons (CC BY). Copyright (2021).

Red-emissive Si QDs have been synthesized using various approaches, including template-assisted growth, Zintl salt oxidation, electrochemical etching, plasma-assisted synthesis, non-thermal plasma decomposition, and pyrolysis of hydrogen silsesquioxane (HSQ) [21,22,75,76,77,78,79,80]. Figure 3a(i) illustrates the non-thermal plasma synthesis of Si QDs, wherein silane (SiH_4_) gas is dissociated under low-pressure radio-frequency (RF) plasma to form hydrogen-terminated silicon nanocrystals. The as-formed species subsequently undergo agglomeration, leading to the formation of Si QDs with controlled size distribution. Maintaining an oxygen-free environment and applying appropriate surface passivation—typically via ligand exchange with alkyl or amine groups—are crucial to prevent oxidation and preserve luminescence efficiency. A liquid precursor, cyclohexasilane (Si_6_H_12_), was used to fabricate Si QDs by plasma-based method, and subsequent density-gradient ultracentrifugation (DGU) in an oxygen-shielded environment improved PLQYs up to 70% at 816 nm (Figure 3a(ii)). Figure 3a(iii) shows the bandgap according to the size of Si QDs, where the recent experimental results are well-matched with computational results [76]. Figure 3b(i) depicts a pyrolytic synthesis strategy utilizing HSQ as a molecular precursor, which undergoes thermal decomposition at ~1100 °C in a reducing atmosphere (95% Ar/5% H_2_), resulting in phase-separated Si QDs embedded within a SiO_2_ matrix. Post-synthesis HF etching releases colloidal Si QDs, followed by ligand exchange to enhance dispersion stability and emission. Conventional cage-type HSQ has been largely replaced by polymeric HSQ precursors, which can be synthesized via one-pot hydrolysis–condensation of silane monomers and decyl-termination by thermal hydrosilylation (Figure 3b(ii,iii)). It reduces precursor cost by 380-fold while achieving high yields (~90%) [78]. The HSQ-based synthesis exhibits highly tunable PL characteristics depending on the precursor structure and surface ligands. For instance, PLQY values of 15%, 37%, and 77% have been reported at emission wavelengths of 743 nm, 867 nm, and 740 nm, respectively, using dodecyl or decyl-based surface ligands [78,79,80].

#### 2.2.3. Germanium (Ge) QDs

Figure 3c(i) illustrates an epitaxial synthesis approach for Ge QDs utilizing molecular beam epitaxy (MBE), in which high-purity Ge atoms are deposited onto Si(001) substrates under ultra-high vacuum conditions. Lithographically defined pits on the substrate surface act as preferential nucleation sites, guiding the spatially ordered self-assembly of Ge QDs via the strain-driven Stranski–Krastanov (S–K) growth mode. The classical concept of forming an epitaxial monolayer is proposed in three stages [81]. Initially, a supersaturated adsorbate of Si atoms (“sea” of Si adatoms) is formed at the substrate surface, and nucleation of 2D islands occurs at the centers. The supersaturation near each island decreases, but the islands do not yet interact with each other, since the diffusion-source fields do not yet overlap. As growth proceeds and the diffusion-source fields begin to overlap, Ostwald ripening initiates and continues until the number of Si adatoms falls below monolayer coverage [81,82]. On the other hand, to form 3D islands (i.e., dots), a supersaturated “sea” of Ge adatoms forms on the surface of an underlying thin Ge layer, known as the wetting layer. Unlike the classical theory, the nucleation of 3D islands (specifically hut-shaped clusters) is triggered by the relaxation of elastic strain. As growth continues, two distinct shapes—huts and domes—emerge and can coexist depending on their specific volumes and energetic advantages. The Ge atoms flow toward the more energetically favorable shapes, while this resembles Ostwald ripening, it often results in a bimodal distribution rather than a single peak. In a closed system without a continuous supply of Ge atoms, island growth becomes self-limited, enabling the formation of spatially confined, size-controlled monolayers, such as dot-like structures [83,84,85]. This site-controlled epitaxy enables precise regulation of dot positioning, lateral dimensions, and areal densities—typically achieving dot sizes below 20 nm and densities in the range of 2 × 10^10^ to 2 × 10^11^ cm^−2^ [23]. The resulting Ge QDs, confined by (105) facets, form elongated hut-shaped structures (Figure 3c(ii)) and exhibit high structural uniformity, making them suitable for integration into silicon-based photonic devices [23]. Periodically and three-dimensionally ordered Ge QDs are grown on Si(001) substrates by MBE. The substrates are pre-patterned with two-dimensional hole arrays using extreme ultraviolet interference lithography (EUV-IL) and reactive ion etching (RIE), which enables highly ordered Ge QD arrays (~3.6 × 10^7^ dots). The atomic force microscopy (AFM) results and the corresponding histogram indicate the narrow size distribution in both diameter and height (Figure 3c(iii,iv)) [86].

Another representative approach for synthesizing Ge QDs is the colloidal hot-injection method [87,88,89]. Early studies report solution–phase routes, using strong reducing alkali metal or organoalkali reagents to convert GeCl_4_ to Ge. For example, a reaction between GeCl_4_ and lithium naphthalide in tetrahydrofuran (THF) leads to 3D growth of Ge nanoparticles by spontaneous reduction in all four chlorine atoms. A subsequent addition of CH_3_SiCl results in the formation of CH_3_Si radicals under the reaction of the CH_3_SiCl with the excess lithium naphthalide, thereby terminating dangling bonds of Ge [85]. Instead of using organoalkali reagents, the reaction of GeCl_4_ with phenyl–GeCl_3_, with a fine dispersion of NaK alloy in heptane, has been proposed to obtain amorphous Ge nanoparticles [44]. Crystallization occurs by heating the mixture in a sealed pressure vessel at 270 °C for 24–48 h, which produces amorphous Ge nanoparticles with average sizes of 6, 11, and >20 nm. Recent research indicates that GeI_2_ undergoes thermal decomposition in a coordinating solvent mixture composed of oleylamine (OAm), trioctylphosphine (TOP), and octadecene (ODE) (Figure 3d(i)). OAm acts as both a reducing agent and a coordinating ligand, while at elevated temperatures (~260–300 °C) the ODE reacts with surface Ge–H bonds through hydrogermylation, replacing OAm and forming covalently bonded Ge–C-terminated surfaces. The addition of TOP further promotes nanocrystal growth by modulating the decomposition kinetics of GeI_2_ (Figure 3d(ii)). The ^1^H NMR spectra indicate that, in the presence of ODE, the Ge QDs no longer exhibit the characteristic signals corresponding to the OAm double bond (Figure 3d(iii)). This suggests that ODE binds to the Ge QD surface through the double bond via hydrogermylation [24]. Fabrication of size-controlled, vertically stacked SiGe QDs has been enabled in a pit-patterned Si substrate, prepared by electron beam lithography and RIE (Figure 3e(i,ii)). The radii of each pit range from 75 nm to 100 nm, which yield pit densities ranging from 4.16 × 10^8^ cm^−2^ to 8.65 × 10^8^ cm^−2^. The sample is etched to a uniform depth of 50 nm to allow for single QD nucleation in each pit during MBE growth, which is done by electron beam evaporation of Si and Ge. The faceted pits act as nucleation sites for the growth of SiGe QDs. Figure 3e(iii,iv) displays an AFM image and corresponding inclination angle map of the single QD arrays (noted as RefA) from pre-patterned areas, which shows excellent ordering in registry [90,91].

## 3. Strategies for Achieving Highly Efficient Red-Emissive QDs

### 3.1. Carbon- or Graphene-Based QDs

#### 3.1.1. Heteroatom Doping

**Figure 4:**
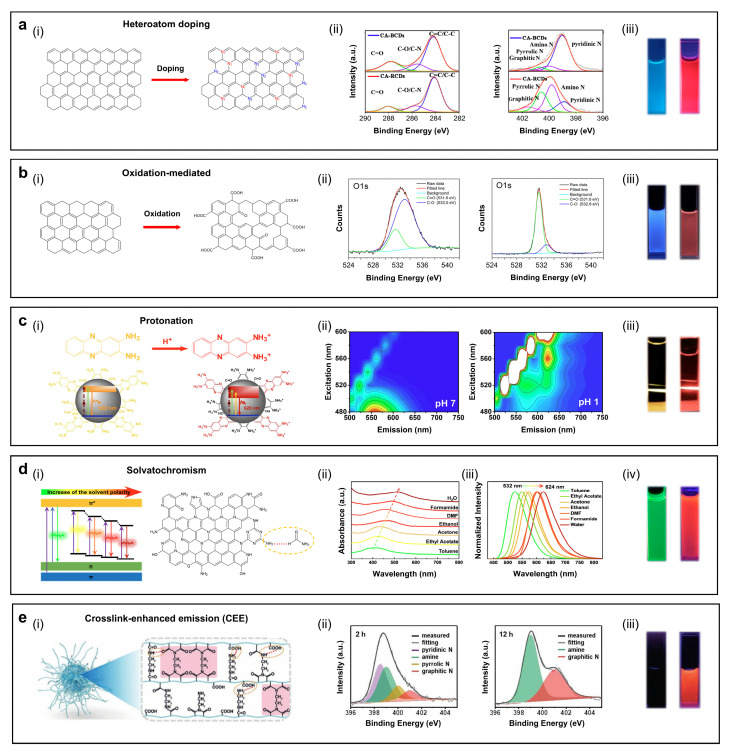
(**a**–**e**) Five representative strategies for achieving red emission in GQDs or CDs. (**a**) Heteroatom doping (**i**), high-resolution X-ray photoelectron spectroscopy (XPS) C 1s (**left**) and N 1s (**right**) spectrum (**ii**), and digital images under UV irradiation (**iii**) of blue-emissive CDs (CA-BCDs) and red-emissive CDs (CA-RCDs) synthesized using citric acid (CA), before (**left**) and after (**right**) nitrogen doping, respectively; reproduced from [63], with permission from Elsevier. Copyright (2022). (**b**) Oxidation (**i**), high-resolution XPS O 1s spectrum (**ii**), and digital images (**iii**) of CDs before (**left**) and after (**right**) oxidation, respectively; reproduced from [26], with permission from the American Chemical Society. Copyright (2016). (**c**) Protonation (**i**), normalized 3D fluorescence (FL) spectra (**ii**), and digital images under UV irradiation (**iii**) of CDs under pH 7 (**left**) and pH 1 (**right**), respectively; reproduced from [64], with permission from Springer Nature. Copyright (2021). (**d**) Solvatochromism (**i**), UV–vis absorption spectra (**ii**), normalized PL spectra (**iii**) of R-GQDs dispersed in various solvents with different polarities, and digital images upon UV irradiation of R-GQDs (**iv**) dispersed in toluene (**left**) and in water (**right**); reproduced from [92], with permission from Elsevier. Copyright (2021). (**e**) Crosslink-enhanced emission (CEE) (**i**), high-resolution XPS N 1s spectrum (**ii**), and digital images of UV irradiation (**iii**) of R-PDs prepared with different heating times of 2 h (**left**) and 12 h (**right**). Reproduced from [93,94], with permission from the Royal Society of Chemistry. Copyright (2018), Wiley–VCH. Copyright (2019).

Figure 4a(i) depicts a simplified model of heteroatom doping of GQDs. Incorporation of heteroatoms into the basal plane of GQDs or into the mixed sp^2^–sp^3^ framework of CDs perturbs the π-electron system and narrows the bandgap [25,59,60,61,63,66,92]. Nitrogen (N) doping, for example, introduces pyridinic, pyrrolic, and graphitic N species. Pyridinic N donates a lone electron pair into the π-electron system, which stabilizes localized electronic states. Pyrrolic N represents five-membered rings where N is embedded. This extends π-conjugation and generates mid-gap states in the intrinsic bandgap. Graphitic N is formed by the substitution of C atoms within the graphene lattice, which redistributes π-electron density [63]. These effects enhance π-electron delocalization and shift the emission of GQDs toward the red spectral region. Figure 4a(ii) shows high-resolution XPS C 1s (left) and N 1s (right) spectra that support changes in chemical bonding after N doping. In the C 1s spectra, the relative intensity of the C–C/C=C component decreases, whereas C–O- and C–N-related peaks become more pronounced. This indicates the formation of N-containing functional groups within the graphene framework. In the N 1s spectra, XPS signals of distinct contributions assigned to pyridinic, pyrrolic, and graphitic N appear. This confirms successful doping of N species into GQDs or CDs and is consistent with the proposed bandgap-narrowing mechanism [63]. Figure 4a(iii) shows digital images of the samples under UV irradiation before and after N doping [63].

#### 3.1.2. Oxidation

Controlling the oxidation level of GQDs or CDs represents another effective approach to reduce bandgaps (Figure 4b(i)) [26,55]. The blue-emissive CDs synthesized from carbon-rich precursors undergo a post-oxidation treatment. During the oxidation, the surface of CDs becomes enriched with oxygen functional groups, including hydroxy (-OH), carbonyl (-C=O), and carboxyl (-COOH) moieties, which is clearly evidenced by the high-resolution XPS O1s spectra in Figure 4b(ii) [26]. After oxidation, the intensity of overall O 1s signals increases, and the relative contributions of the carbonyl and carboxyl components become more pronounced (left panel in Figure 4b(ii)). It indicates the formation of highly oxidized surface states, which introduce additional surface trap states, thereby narrowing the bandgap. A pronounced redshift in emission from blue to deep-red was visualized by the digital images of the CDs before and after oxidation, as shown in Figure 4b(iii) [26].

#### 3.1.3. Protonation

Figure 4c(i) illustrates a protonation-induced bandgap modulation mechanism in *o*-phenylenediamine (OPD)-derived CDs bearing surface 2,3-diaminophenazine (2,3-DAPN) fluorophores [13]. During synthesis, OPD is carbonized to form the carbon core, while partial oxidation generates 2,3-DAPN molecules that are anchored on the CD surface as emissive units. Under acidic conditions, the amino groups (–NH_2_) of surface-bound 2,3-DAPN are protonated to –NH_3_^+^, altering the electronic structure and reducing the effective bandgap of the CDs. As shown in Figure 4c(ii), the PL peak shifts from yellow emission at ~550 nm (at pH 7) to red emission at ~620 nm under strong acidic conditions (at pH 1), indicating protonation-induced stabilization of lower-energy excited states and enhanced long-wavelength emission. Consistent with this behavior, protonation leads to an upward shift in the HOMO energy level from 7.25 eV to 8.15 eV, thereby reducing the energy gap between the HOMO and LUMO levels from 2.53 eV to 2.38 eV [13]. Figure 4c(iii) further confirms this transition by digital images under UV irradiation, showing a distinct color change from yellow to red upon protonation [64].

#### 3.1.4. Solvatochromism

Figure 4d(i) illustrates a solvatochromic emission model for red-emissive GQDs (R-GQDs) synthesized from *p*-phenylenediamine and melamine via mild hydrothermal treatment [59,92]. The resulting R-GQDs exhibit a uniform size distribution, excitation-independent emission, and good photostability across a wide range of solvents. Solvatochromism describes the change in absorption or emission wavelength that occurs when the fluorophore interacts with solvents of different polarities. In R-GQDs, polar solvents preferentially stabilize the excited states relative to the ground state, effectively reducing the energy gap between them and allowing the emission color to be tuned from blue–green to red. As the solvent polarity increases, this stabilization leads to a delayed electronic transition and a longer excited-state lifetime, which in turn enhances emission from lower-energy states and strengthens the red component of the fluorescence. Figure 4d(ii) shows that the main absorption band of the R-GQDs gradually shifts to longer wavelengths with increasing solvent polarity, consistent with the stabilization of the excited states [92]. Figure 4d(iii) further reveals that the PL peak moves from 532 nm in a low-polarity solvent to 624 nm in a highly polar solvent [92]. Digital images under UV illumination further confirm this solvatochromic behavior, with R-GQDs displaying greenish emission in toluene and bright red emission in water (Figure 4d(iv)) [92].

#### 3.1.5. Crosslink-Enhanced Emission (CEE)

The PDs fabricated from non-conjugated polymers typically do not exhibit intrinsic luminescence as free molecular motion facilitates non-radiative energy dissipation. However, the introduction of crosslinks immobilizes molecular motion, activating emission through the crosslink-enhanced emission (CEE) mechanism. Restriction of intermolecular movement minimizes vibrational and rotational relaxation pathways, thereby promoting radiative recombination and enabling red emission [93,94]. Here, red-emissive PDs (R-PDs) are synthesized via a one-pot modulated polymerization of *p*-phenylenediamine (pPD) with FeCl_3_ catalyst in water, followed by heating at 80 °C for 0–36 h in a sealed tube. This process generates N-rich surface states by covalent crosslinks among pPD oligomers, which enables CEE-driven red emission at *λ*_em_ ≈ 600 nm. Figure 4e(i) illustrates immobilization of sub-fluorophores within dense polymer networks through crosslinking. This enhances electronic coupling and restricts non-radiative decay [93]. Figure 4e(ii) shows high-resolution XPS 1 1s spectra, which indicate the evolution of N-related functional groups over reaction time. At 2 h, amine-type N (~399 eV) dominates, whereas at 12 h, a peak indicating graphitic N (~401 eV) increases. At the same time, the C=N and C=O components (~288.7 eV) appear in the C 1s spectrum. Figure 4e(iii) shows that the precursors exhibit no emission at initial states, while the R-PDs display strong red emission under UV irradiation, directly confirming that crosslinking and N reconfiguration activate CEE-mediated red emission [94]. Synthesis and properties of red-emissive, GQDs or CDs are summarized in Table 1.

### 3.2. Inorganic Core-Shell QDs

#### 3.2.1. Indium Phosphide (InP)

**Figure 5:**
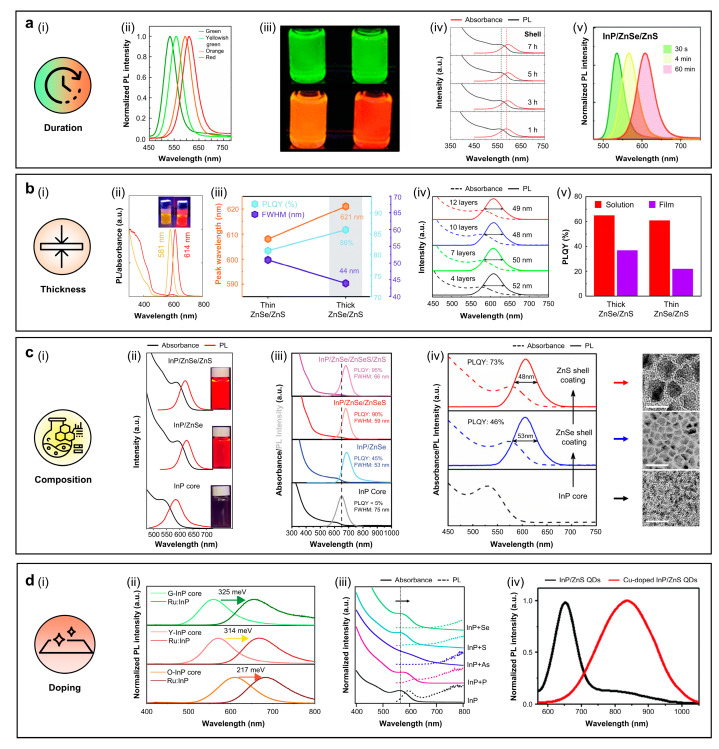
(**a**–**d**) Four representative strategies for achieving efficient red emission in indium phosphide (InP) QDs. (**a**) The duration of the InP core growth (**i**), PL spectra (**ii**) and digital images under UV irradiation (**iii**) of InP/ZnS QDs, synthesized with InP core growth times of 0.5 min (green), 1 min (yellowish green), 2 min (orange), and 5 min (red), with a constant shelling time of 7 h and with absorption/PL spectra of orange InP/ZnS QDs synthesized with shelling times of 1–7 h (**iv**). Reproduced from [16], with permission from Springer Nature. Copyright (2013), and normalized PL spectra of InP/ZnSe/ZnS QDs, synthesized with InP core growth times of 30 s, 4 min, and 60 min (**v**). Reproduced from [68], with permission from the Royal Society of Chemistry. Copyright (2022). (**b**) Controlling the thickness of the core or shell materials (**i**), absorbance/PL spectra, with digital images under UV irradiation (**ii**). Reproduced from [72], with permission from the Royal Society of Chemistry. Copyright (2024), peak wavelength, PLQY, and FWHM (**iii**). Reproduced from [68], with permission from the Royal Society of Chemistry. Copyright (2022) of thin (orange) and thick (red) InP/ZnSe/ZnS QDs, absorbance/PL spectra of InP/ZnSe/ZnS QDs with different ZnS layers (**iv**), and absolute PLQYs of thick and thin InP/ZnSe/ZnS QDs (**v**). Reproduced from [69], with permission from the American Chemical Society. Copyright (2018). (**c**) The composition (**i**), absorbance/PL spectra of InP core, InP/ZnSe, and InP/ZnSe/ZnS QDs, with digital images under UV irradiation (**ii**). Reproduced from [74], with permission from Wiley–VCH. Copyright (2022), InP core, InP/ZnSe, InP/ZnSe/ZnSeS, and InP/ZnSe/ZnSeS/ZnS QDs (**iii**). Reproduced from [8], with permission from Wiley–VCH. Copyright (2023), InP core, InP/ZnSe, and InP/ZnSe/ZnS QDs (**left panel**) with corresponding TEM images (**right panel**) (**iv**). Reproduced from [69], with permission from the American Chemical Society. Copyright (2018). (**d**) Doping (**i**), PL spectra of Ru:InP QDs with green-emitting (G-InP), yellow-emitting (Y-InP), and orange-emitting (O-InP) cores and maximum PL shift after Ru doping at different Ru/In ratios (**ii**). Reproduced from [107], with permission from the American Chemical Society. Copyright (2023), absorbance/PL spectra of native, P, As, S, and Se-treated InP QDs (**iii**). Reproduced from [108], with permission from AIP Publishing. Copyright (2021), and PL spectra of InP/ZnS and Cu-doped InP/ZnS QDs (**iv**). Reproduced from [109] with permission from the American Chemical Society. Copyright (2019).

Figure 5a illustrates one of the representative strategies for achieving red-emissive InP QDs, where the emission wavelength is tuned by controlling the core growth duration (Figure 5a(i)). InP cores grown for 0.5, 1, 2, and 5 min, followed by 7 h of ZnS shelling, yield InP/ZnS core–shell QDs of gradually increasing size (from ~2.7 to 3.1 nm), with corresponding emission colors of green, yellowish green, orange, and red. Due to the quantum confinement effect, the PL peak positions of these QDs appear at 532 nm (green), 558 nm (yellowish green), 593 nm (orange), and 612 nm (red) (Figure 5a(ii)). The digital image under UV illumination (Figure 5a(iii)) visually confirms this emission color change. The shell thickness also influences the PL characteristics of InP/ZnS QDs. As the shelling time increases from 1 h to 7 h, the PL peak is gradually red-shifted from 590 nm to 596 nm, indicating continuous ZnS shell growth (Figure 5a(iv)). Furthermore, the PLQY improves from approximately 30% to 53%, which might be attributed to enhanced exciton wave-function leakage into the shell region with increasing shell thickness [16]. This tendency is in line with the strategies summarized in Table 2, where gradient temperature-rise methods enable the growth of larger QDs (~12 nm) while maintaining high PLQYs (~90%) and narrow FWHM (37 nm), thereby indicating improved crystallinity and surface passivation [69]. Additionally, InP QDs synthesized using InBr_3_ as a precursor show broadening of PL intensity and FWHM, increasing from 37 nm (green) to 51 nm (red) with longer core-growth times. The average core diameters are 2.2 nm (30 s), 2.6 nm (4 min), and 3.1 nm (60 min), and the corresponding InP/ZnSe/ZnS heterostructures exhibit emission peaks at 536 nm (green), 566 nm (yellow), and 608 nm (red) (Figure 5a(v)) [68].

Figure 5b presents another strategy for achieving efficient red-emissive InP QDs by controlling both the core size and bilayer shell thickness (Figure 5b(i)). Figure 5b(ii) shows the absorbance and PL spectra of two different-sized InP/ZnSe/ZnS core–shell QDs. With increasing InP core size (from 2.4 nm to 3.1 nm) and ZnSe/ZnS shell thickness (from 1.1/1.8 nm to 1.2/1.9 nm), the PL peak red-shifts from 581 nm to 614 nm, with clear emission color transition [72]. In a separate study employing InBr_3_ instead of In(OAc)_3_ as the indium precursor, InP cores grown for 60 min were coated with ZnSe/ZnS shells of two different thicknesses (thin, 1.7 nm, and thick, 2.5 nm). The results in Figure 5b(iii) show that the PLQY increased from 81% to 86%, the FWHM decreased from 51 nm to 44 nm, and the PL peak wavelength is redshifted from 608 nm to 621 nm in QDs with thick shells compared to thin shells [68]. Specifically, varying the number of ZnS shell deposition cycles from 1, 3, 5, to 7 resulted in shell thicknesses of 9.4, 11.6, 13.5, and 15.0 nm, corresponding to 4, 7, 10, and 12 monolayers, respectively. Among these, the QDs with a 10-monolayer (13.5 nm) ZnS shell exhibited the most effective surface passivation, achieving the highest PLQY of 73% and a narrowed FWHM of 48 nm (Figure 5b(iv), [69]). Further shell growth to 12-monolayers (15 nm) led to a decrease in PLQY, likely due to the introduced dislocations and low-angle grain boundaries.

The importance of shell structuring is further evidenced by the poor properties of InP cores without shells or with only ZnSe shells, which typically exhibit PLQYs less than 50% and broad FWHM around 70 nm due to non-radiative recombination at surface defects [68,103]. Optimization of core/double-shell architectures, such as InP/ZnSe/ZnS, results in a high PLQY above 90%, and narrow FWHM (<40 nm) in the 610–630 nm emission range [13,14,71,72,73]. Direct deposition of ZnS shells on InP cores often results in lattice mismatch-induced strain and defect formation. To reduce this, the ZnSe intermediate shell is coated before the ZnS shell, serving as a structural buffer layer [104]. For instance, controlling the thickness of the ZnSe/ZnS double-shell results in an emission redshift from 606 nm to 611 nm [104]. Moreover, sufficiently thick ZnS shells act as spacers that suppress Förster resonance energy transfer (FRET) between neighboring QDs, thereby reducing efficiency losses that occur from interparticle interactions [69]. As shown in Figure 5b(v), the PLQYs of thick-shelled QDs exhibit only minor decreases when transitioning from solution to solid film, demonstrating the effective suppression of non-radiative FRET and enhanced exciton recombination efficiency [69]. These results emphasize that precise core/shell engineering, particularly with optimized ZnSe/ZnS double-shell structures, is essential to achieve bright and spectrally pure red emission [13,14,68,69,71,72,73,103,104,105,106].

Distinct changes in optical properties of QDs are observed when the structure evolves from core to core/shell to core/double-shell architectures (Figure 5c(i,ii)) [74]. The formation of InP/ZnSe/ZnS core/double-shell structure is confirmed by elemental analysis that In and phosphorus were concentrated in the cores, whereas Zn and S were predominantly distributed in the outer ZnSe/ZnS shells [15]. Spectroscopic measurements further reveal that ZnS overcoating significantly enhanced PLQY, though in some cases it induces spectral shifts, depending on the ratio of precursor (Figure 5c(iii)) [8]. Charge transfer between the InP core and the ZnSe inner shell, followed by enhanced carrier confinement upon ZnS outer shell deposition, leads to slight PL redshifts and narrowing of FWHM (Figure 5c(iv)) [69]. Corresponding TEM images support these results by showing structural evolution across core, single-shell, and double-shell architectures (right panel in Figure 5c(iv)).

Heteroatom doping in InP QDs enables controlled bandgap narrowing and suppression of surface traps without degrading PLQYs or structural stability (Figure 5d(i)). For example, ruthenium (Ru)-doped InP core QDs exhibit a PL redshift according to the Ru/In ratio (Figure 5d(ii)). For G-InP cores, a maximum redshift of 325 meV is obtained at a Ru/In ratio of 0.005. The Y-InP and O-InP cores show maximum shifts of 314 meV and 217 meV at a Ru/In ratio of 0.01, respectively. These results indicate that Ru-induced mid-gap states located above the valence band maximum act as hole acceptors, which facilitates recombination with electrons in the conduction band. The subsequent reduction in PL shift at higher doping levels is attributed to dopant site saturation and surface oxide formation [107]. Beyond Ru doping, surface anion treatments using precursors such as P(SiMe_3_)_3_ and As(SiMe_3_)_3_ have also been reported to induce red-shifted absorption while simultaneously quenching excitonic PL, owing to reduced electron–hole overlap and band structure modification. Similarly, S or Se treatments induce redshifted trap–state emissions associated with hole trapping by surface P dangling bonds, indicating that the electronic structure can be modulated through surface chemistry without altering the core framework (Figure 5d(iii)) [108]. Among various dopants, Cu yields the most prominent long-wavelength emission. Whereas undoped InP/ZnS QDs exhibit narrow excitonic emission at ~650 nm (FWHM: 65 nm), Cu-doped InP/ZnS QDs show complete suppression of excitonic PL and instead produce a broad dopant-induced emission band centered at 835 nm, with an FWHM of 190 nm (Figure 5d(iv)) [109]. These results highlight the promise of doping strategies to access red-emission, while also underscoring the inherent trade-off between emission tunability and the preservation of high PLQYs and narrow linewidths in heavily doped InP QDs [107,108,109,110,111].

In addition to the four representative strategies described above, other approaches have also been explored to achieve red emission in InP QDs. A variety of synthetic methods, including hot-injection, thermal decomposition, seed-mediated growth, successive ion layer adsorption and reaction (SILAR), and gradient temperature-rise processes, have been employed (Table 2). Among these, the hot-injection method remains the most widely adopted owing to its operational simplicity, rapid nucleation kinetics, and tunability of particle growth [8,16,70,71,72,73,103]. Particle size also plays a critical role in determining the emission wavelength. Typically, smaller QDs with diameters of 3.6–5.3 nm emit in the ~621–650 nm range, whereas larger QDs exceeding 10 nm generate red-shifted emission spanning 680–835 nm [8,74,103,105,110]. However, excessive growth beyond the optimal size often results in broadened emission linewidths (FWHM > 60 nm) and decreased PLQYs due to aggregation or the formation of surface trap states [8,110]. Collectively, these findings demonstrate that, beyond duration, thickness, composition, and doping, both synthetic methodology and particle size control serve as powerful and complementary strategies for tuning and optimizing the red-emissive properties of InP QDs.

**Table 2:** Properties of red-emissive, InP core-based QDs.

Composition	Method	Precursor	Size [nm]	PL Peak [nm]	FWHM [nm]	PLQY [%]	Year	Ref.
InP/ZnSe/ZnSeS/ZnS	Thermal decomposition	InCl_3_, (DMA)_3_P	10.5	680	66	~95	2023	[8]
InP/ZnSe/ZnSeS			8.4	680	59	90	2023	
InP/ZnSe			6.8	683	53	45	2023	
InP	Hot-injection	InCl_3_, (DMA)_3_P	5.3	650	75	~5	2023	
InP/thick-ZnSe/ZnS	Hot-injection	In(Ac)_3_, (TMS)_3_P	8.8	621	44	86	2022	[68]
InP/ZnSe/ZnS	Gradient temperature-rise process	In(PA)_3_, TMSi_3_P	12	614	37	~90	2024	[103]
InP/ZnSe/ZnS	Hot-injection	In(OAc)_3_, (TMS)_3_P	9.3	614	35	95	2024	[72]
InP/ZnSe/ZnS	Thermal decomposition	In(Ac)_3_, (TMS)_3_P	12	614	37	92	2024	[15]
InP/ZnSe/ZnS	Seed-mediated	InBr_3_, P(NEt_2_)_3_	11	620	35	90	2024	[70]
InP/Zn(Se,S)/ZnS	Thermal decomposition	In(Ac)_3_, (TMS)_3_P	10.8	607	45.5	95	2022	[13]
InP/ZnSe/ZnS	Hot-injection	InX_3_, ZnX_2_, (DEA)_3_P (X: Cl, Br or I)	9.3	611	40	~73	2020	[104]
InP/ZnSe/ZnS	Thermal decomposition	In(OAc)_3_, (TMS)_3_P	7.8 ± 0.5	618	42	93 ± 3	2019	[71]
InP/ZnSe/ZnS	Hot-injection, thermal decomposition	InBr_3,_ (DMA)_3_P	-	630	35	~100	2019	[14]
InP/ZnSe/ZnS	Thermal decomposition	In(Ac)_3_, (TMS)_3_P	15	607	48	73	2018	[69]
InP/ZnSe/ZnS	Hot-injection, SILAR	In(OAc)_3_, (TMS)_3_P	~3.6	621	44	50~	2017	[73]
InP/ZnSe	Heat-up method	InCl_3_, (DEA)_3_P	-	621	54	35	2019	[112]
Ru: InP/ZnS	Hot-injection method	InCl_3_, (TMS)_3_P	5.83 ± 0.81	655	-	77.6	2023	[107]
InP/ZnS:Cu QD	Hot-injection, SILAR	In(Ac)_3_, (TMS)_3_P	3.9 ± 0.4	835	190	40	2019	[109]
InP/ZnSe/ZnS	Gradient temperature-rise process	In(PA)_3_, TMSi_3_P	12	614	37	~90	2024	[103]

#### 3.2.2. Copper–Indium (Cu-In) Chalcogenides

##### Cu/in Ratio

**Figure 6:**
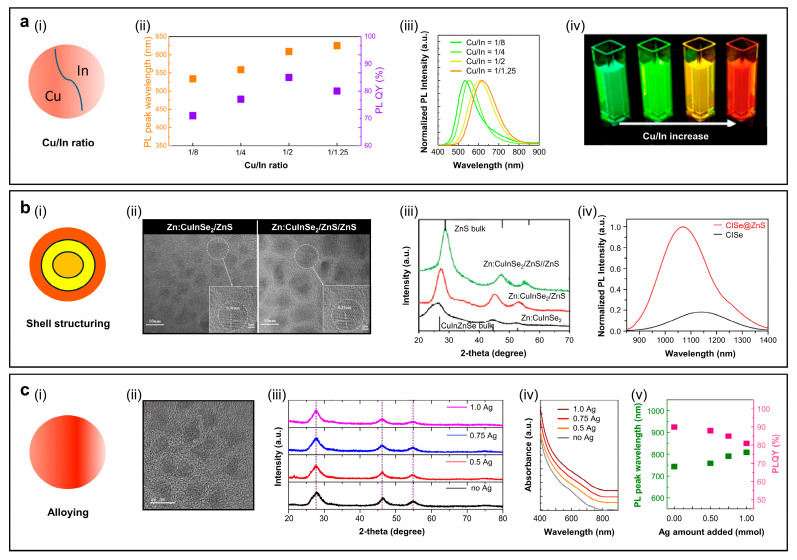
(**a**–**c**) Three representative strategies for achieving efficient red emission in copper–indium (Cu-In) core-based QDs. (**a**) The Cu/In ratio (**i**), PL peak wavelength and PLQY (**ii**), normalized PL spectra (**iii**), and digital images under UV irradiation (**iv**) of CIS/ZnS QDs synthesized with Cu/In molar ratios of 1/8, 1/4, 1/2, and 1/1.25 under the same core growth conditions. Reproduced from [113], with permission from the American Chemical Society. Copyright (2019). (**b**) The shell structuring (**i**), HRTEM images of Zn:CuInSe_2_/ZnS (**left**) and Zn:CuInSe_2_/ZnS//ZnS (**right**) core/shell QDs (**ii**), XRD patterns of Zn:CuInSe_2_ core, Zn:CuInSe_2_/ZnS, and Zn:CuInSe_2_/ZnS//ZnS core/shell QDs (**iii**), and normalized PL spectra of CISe and CIS@ZnS QDs (**iv**). Reproduced from [18], with permission from Optica Publishing Group. Copyright (2022). (**c**) The scheme for alloying (**i**), HRTEM image of Ag-alloyed CIS (ACIS) core-based QDs (**ii**), XRD patterns (**iii**), absorbance (**iv**), and PL peak wavelength and PLQY (**v**) of ACIS core-based QDs alloyed with varying Ag amounts of 0, 0.5, 0.75, and 1.0 mmol. Reproduced from [113], with permission from the American Chemical Society. Copyright (2019).

Off-stoichiometric control of the Cu/In ratio in I-III-VI group chalcogenide QDs is a key strategy to enable precise control over emission color from green to red by modulating the density of donor–acceptor pair (DAP) states [17,41,113,114]. Cu enrichment narrows the bandgap and promotes defect-related electronic transitions [17,41,115,116], while Cu deficiency widens the bandgap due to the weakened repulsion between Cu *d* and S (or Se) *p* orbitals. As shown in Figure 6a(i), adjusting the Cu/In ratio in CIS QDs significantly affects both the PL peak wavelength and PLQY [17]. Increasing the Cu/In ratio in the CIS/ZnS core/shell QDs results in a pronounced PL redshift (from ~520 to ~640 nm) and enhances the PLQY (up to ~95%) under identical core growth conditions (180 °C, 15 s) (Figure 6a(ii)) [113]. The normalized PL spectra in Figure 6a(iii) confirm that higher Cu content promotes deeper defect-related states. The resulting DAP recombination enables sub-bandgap PL emission through Cu-related trap states [17,41,113]. A digital image of four CIS/ZnS QDs with Cu/In ratios of 1/8, 1/4, 1/2, and 1/1.25 under UV exposure further supports these results, showing a clear emission color transition from green to red (Figure 6a(iv)) [113].

##### Core–Shell Structuring

A widely adopted strategy to improve the PLQY and stability of red/NIR-emissive CIS QDs is the formation of core/shell structures [17,18,41,113,115,116,117,118,119]. Figure 6b(i) illustrates a conventional double-shell architecture [18]. High-resolution transmission electron microscopy (HRTEM) images in Figure 6b(ii) confirm the formation of Zn:CuInSe_2_/ZnS and Zn:CuInSe_2_/ZnS//ZnS QDs, with the latter exhibiting more uniform and crystalline lattice fringes [18]. As shown in Figure 6b(iii), X-ray diffraction (XRD) peaks shift toward those of bulk ZnS with additional ZnS shell layers, indicating enhanced crystallinity and improved shell growth [18]. In Figure 6b(iv), the PL intensity of CISe@ZnS is significantly enhanced compared to bare CISe cores [18]. The PL peak is blue-shifted from ~1140 nm in CISe QDs to ~1070 nm in CISe@ZnS QDs due to the shrinkage of the effective size of the CISe core after ZnS coating [18,113]. This highlights the effective passivation of surface defects by the ZnS shell and suppression of non-radiative recombination.

##### Alloying

Alloying with Ag is an effective method to tailor the optical properties of CIS QDs (Figure 6c(i)). The HRTEM in Figure 6c(ii) confirms that the lattice constant is ~5.4 nm, which remains intact after Ag incorporation (1.0 mmol). XRD patterns in Figure 6c(iii) show that the characteristic diffraction peaks at 2θ = 27.9°, 46.4°, and 54.8° are retained for all Ag contents (0–1.0 mmol), without the emergence of secondary phases. This indicates that the chalcopyrite CIS crystal structure is preserved during Ag alloying [113]. The absorption spectra in Figure 6c(iv) reveals a bathochromic shift with increasing Ag content, which implies that bandgap narrowing is attributed to enhanced electronic delocalization or defect state modification. Resulting QDs exhibit PL redshift from 744 nm to 806 nm, although the PLQY decreases beyond a certain Ag concentration (Figure 6c(v)).

In summary, the Cu/In ratio tuning modulates the electronic structure and recombination pathways, shell engineering effectively passivates surface states to boost PLQY, and alloying with Ag offers bandgap engineering for emission tunability. These strategies, individually or in combination, provide a versatile toolbox for the rational design of highly efficient, red-emitting CIS-based quantum dots [17,18,41,113,114,115,116,117,118,119]. Table 3 summarizes composition, synthesis method, precursors, and several key optical properties of CIS QDs.

#### 3.2.3. Silver–Indium (Ag-In) Chalcogenides

##### Shell Structuring

**Figure 7:**
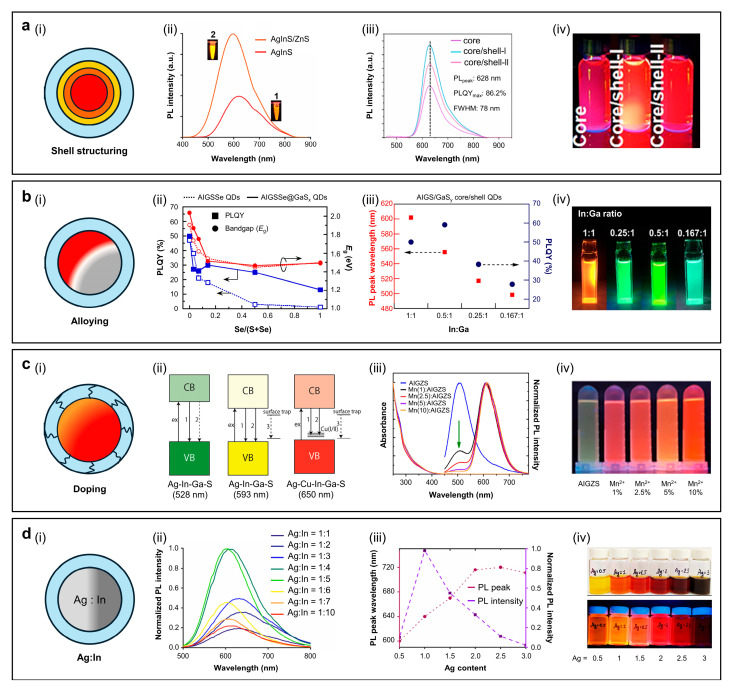
(**a**–**d**) Strategies for achieving efficient red emission in silver–indium (Ag-In) core-based QDs. (**a**) The shell structuring (**i**), PL spectra of AgInS and AgInS/ZnS (**ii**), PL spectra (**iii**), and digital image under UV irradiation (**iv**) of Ag-In QDs, with different compositional InS_x_ shell structuring: Zn_0.32_Ag_1_In_1.37_Ga_0.34_ (core), Zn_0.30_Ag_1_InS_1.39_Ga_0.32_/InS_0.15_ (core/shell-I), and Zn_0.30_Ag_1_InS_1.33_Ga_0.35_/InS_0.94_ (core/shell-II) QDs. Reproduced from [123,124], with permission from the American Chemical Society. Copyright (2023, 2024). (**b**) The alloying (**i**), PLQY and bandgap (*E*_g_) of AIGSSe QDs and AIGSSe@GaS_x_ QDs (**ii**), PL peak wavelength and PLQY (**iii**), and digital image under UV irradiation (**iv**) of AIGS/GaS_y_ QDs, depending on the In:Ga ratio (**iv**). Reproduced from [125,126], with permission from the Royal Society of Chemistry. Copyright (2021), the American Chemical Society. Copyright (2021). (**c**) The doping (**i**), energy band diagram of Ag-In-Ga-S QDs, with high Ga content (green) and low Ga content (yellow), and Ag-Cu-In-Ga-S QDs (red) (**ii**), PL spectra (**iii**), and digital image under UV irradiation (**iv**) of AIGZS QDs with different Mn doping concentration. Reproduced from [127,128], with permission from the Bulletin of the Chemical Society of Japan, the American Chemical Society. Copyright (2021), the American Chemical Society. Copyright (2021). (**d**) Ag:In composition control (**i**), PL spectra (**ii**), PL peak wavelength and normalized PL intensity (**iii**), and digital image under UV irradiation (**iv**) of Ag-In QDs at varying Ag:In ratio. Reproduced from [123], with permission from the American Chemical Society. Copyright (2024).

AIS and AISe nanocrystals inherently exhibit a high density of intrinsic defects, including silver vacancies (*V*_Ag_), antisite defect of indium at silver sites (In_Ag_), and chalcogen vacancies (*V*_S_ or *V*_Se_). These defects degrade chemical stability and introduce non-radiative recombination pathways, thereby reducing PLQY. A widely adopted strategy to mitigate these effects involves forming core/shell structures for surface passivation. Effective shell materials require a wider bandgap than the core to ensure carrier confinement and a minimal lattice mismatch to reduce interfacial strain and defect formation. Among available options, ZnS and ZnSe are commonly used due to their Cd-free composition, large bandgaps (~ 2.7 eV), and high chemical stability. Growth of a ZnS shell on Ag-In cores induces notable changes in PL, including a ~30 nm blue shift of the emission peak and an intensity enhancement of about 60% compared to bare AIS cores (Figure 7a(ii)) [123]. In addition to ZnS, the formation of an InS_x_ shell also affects the emission properties of Ag-In core-based QDs. InI_3_ and dimethylthiourea (DMTU) have been used as InS_x_ precursor, producing core, core/shell-I, and core/shell-II compositions of Zn_0.32_Ag_1_In_1.37_Ga_0.34_, Zn_0.30_Ag_1_InS_1.39_Ga_0.32_/InS_0.15_, and Zn_0.30_Ag_1_InS_1.33_Ga_0.35_/InS_0.94_, respectively. The PL spectrum shows a peak at 628 nm with an FWHM of 78 nm, which remains unchanged after shell coating (Figure 7a(iii)). However, the PLQY is significantly enhanced from 27.0% (core) to 86.2% (core/shell-I) and 61.2% (core/shell-II), attributed to effective passivation of *V*_S_ by sulfur, and substitution of *V*_Ag_ with In^3+^ [124]. Under UV illumination (365 nm), all three samples display bright red emission (Figure 7(iv)), with the CIE coordinates located in the red region at (0.667, 0.332) [124].

##### Alloying

Alloying represents an effective strategy to modulate the optical properties of Ag-In core-based QDs by partially substituting Ag^+^ and In^3+^ with other cations (e.g., Ga^3+^, Zn^2+^, Cu^2+^, or Cd^2+^) or S^2-^ with other anions (e.g., Se^2-^, or Te^2-^), thereby forming ternary or quaternary alloyed structures. These and other types of alloying enable PL to redshift toward the red or NIR spectral regions. Figure 7b(i) illustrates the alloyed structure of the QDs. Alloying processes, for example, the direct co-introduction of Ag, In, and Ga precursors during synthesis, or post-synthetic cation exchange following core formation, enable partial substitution of In^3+^ with Ga^3+^. Substitution of these group-III elements strengthens chemical bonding within the lattice, effectively reducing defects and stabilizing the crystal structure. However, the bandgap widens with an increase in bond strength resulting from lattice mismatch, which induces a PL blueshift. Consequently, the alloying process reduces FWHM and suppresses nonradiative recombination by mitigating lattice mismatch and effective passivating both surface and internal defects.

Figure 7b(ii) shows that alloying S with Se in AIS QDs modulates the bandgap via a bowing effect, producing a redshift up to Se/(S+Se) = 0.5, followed by a blueshift at higher Se content. GaSₓ shell coating improves the PLQY of Ag-In-Ga-S-Se core/GaS shell (AIGSSE@GaS_x_) QDs (~28%) compared to that of Ag-In-Ga-S-Se core-only (AIGSSe) QDs (~5%) at Se/(S+Se) = 0.5, while the PLQY gradually decreases as increasing Se content due to structural imperfections revealed by XRD results. The XRD patterns indicate tetragonal structures for all compositions but show gradient alloying and lattice strain at Se/(S+Se) = 0.5 and above, along with a weak Ga_2_O_3_ peak (~36°) from partial Ga oxidation. These factors increase defect density and nonradiative recombination, accounting for the reduced PLQY at higher Se contents [125]. Another study reported that decreasing the In content in In–Ga alloyed (AIGS/GaS_y_) QDs initially widens the bandgap, as the reduction in In_2_S_3_ leads to a Ga-rich composition with a larger bandgap at the QD surfaces. Subsequently, the remaining In_2_S_3_ tends to alloy with Ga_2_S_3_ to form a Ga-rich shell, which relaxes lattice strain and slightly weakens the quantum confinement effect.

The PL peak is gradually blue-shifted from 601 nm to 498 nm when the ratio of In:Ga changes from 1:1 to 0.167:1 (Figure 7b(iii)). The PLQY value increases from 50% to 59% when the ratio of In:Ga changes from 1:1 to 0.5:1, while further increasing Ga content introduces defect-related emission that lowers the PLQY from 59% to 27% when the ratio of In:Ga changes from 0.5:1 to 0.167:1 (Figure 7b(iii)). A digital image of four AIGS/GaS_y_ QDs with an In:Ga ratio of 1:1, 0.25:1, 0.5:1, and 0.167:1 under 365 nm excitation supports these results by showing a visible color transition from orange to cyan (Figure 7b(iv)) [126].

##### Doping

The incorporation of a small number of ions serves as another effective strategy to modulate or create new recombination centers for red emission (Figure 7c(i)). Such doping generates additional electronic states within the band structure, thereby tailoring electron–hole recombination pathways. Doping with transition metals, such as Mn or Cr, has been widely explored to generate new radiative centers. Mn doping enables PL via Mn *d*-states, whereas Cu doping modulates donor–acceptor recombination centers by introducing deep donor levels, extending recombination pathways, and inducing red-shifted emission [127,128].

Figure 7c(ii) shows that introducing Ga precursors into AIS QDs yields Ag-In-Ga-S (AIGS) QDs, and subsequent partial substitution of Ag with Cu produces Ag-Cu-In-Ga-S (ACIGS) QDs. This compositional tuning results in a pronounced redshift of the emission peak from 528 to 650 nm. The PL decay profiles of the AIGS/GaS core/shell QDs are analyzed by multi-exponential fitting. The sample with high Ga content (left panel in Figure 7c(ii)) is well fitted by a bi-exponential function, indicating a dominant band–edge recombination. In contrast, the sample with low Ga content (center panel in Figure 7c(ii)) is fitted by a tri-exponential fitting, suggesting an additional long-lived component associated with trap-assisted recombination. As the Ga content decreases, the contribution of this slow decay component increases, resulting in longer average lifetimes. The emission spectra also support this trend. High-Ga-content QDs exhibit a strong band–edge emission centered at 528 nm, while low-Ga-content QDs showed a red-shifted peak at 593 nm dominated by defect-related emission. These results confirm that decreasing Ga content enhances the influence of defect states, leading to red-shifted and longer-lived PL. Cu incorporation induces localized-state emission dominated by self-trapped excitons, originating from transient Cu(II)-related hole localization through strong electron–phonon coupling, which accounts for the observed broadband and multicomponent PL (right panel in Figure 7c(ii)) [127].

In addition to Cu and Ga, Mn doping has also been demonstrated in Ag-In-Ga-Zn-S (AIGZS) QDs. Figure 7c(iii) presents the UV absorption and PL spectra of Mn-doped AIGZS QDs with Mn^2+^ concentrations of 1, 2.5, 5, and 10%. The absence of distinct absorption peaks is a characteristic of multinary QDs, arising from compositional inhomogeneity that broadens vibrational states within the QDs [128]. The Mn-related emission originates from the ^4^T_1_ to ^6^A_1_ radiative transition of Mn^2+^, typically observed when the host bandgap exceeds the Mn^2+^ transition energy, although it may coexist with donor–acceptor transitions. Figure 7c(iv) shows digital images of pristine AIGZS QDs and Mn-doped (1–10%) AIGZS QDs under UV illumination [128].

##### Ag/in Ratio

Compositional tuning of AIS QDs offers a route for red emission (Figure 7d(i)). Lowering the silver content increases Ag vacancy defects that act as shallow acceptor states and promotes donor–acceptor pair recombination [123]. However, Ag-deficient compositions also generate more surface traps, necessitating core/shell structures for effective passivation. The PL spectra in Figure 7d(ii) shows that increasing the Ag:In molar ratio up to 1:5 induces a blueshift of the PL maximum from 647 to 605 nm, accompanied by a gradual increase in PL intensity, which reaches its maximum at Ag:*x*In = 1:5. To extend the emission of AIS QDs toward red and NIR, samples are synthesized with an Ag:In:S ratio of *x*:5:5 while varying the Ag content (*x* = 0.5–3). Figure 7d(iii) shows that increasing Ag redshifts the PL peak from 600 to 710 nm, with maximum intensity at Ag:In:S = 1:5:5, followed by a decrease at higher Ag concentrations (*x* > 1). This modulation arises from bandgap narrowing due to hybridization of Ag 4d and S 3p orbitals, which raises the valence band. Figure 7d(iv) shows digital images of AgInS core QDs with different Ag contents under UV excitation (λ_exc_ = 365 nm). Table 4 summarizes composition, synthesis method, precursors, and several key optical properties of Ag-In core-based QDs.

### 3.3. Silicon or Germanium QDs

#### 3.3.1. Common Strategies

**Figure 8:**
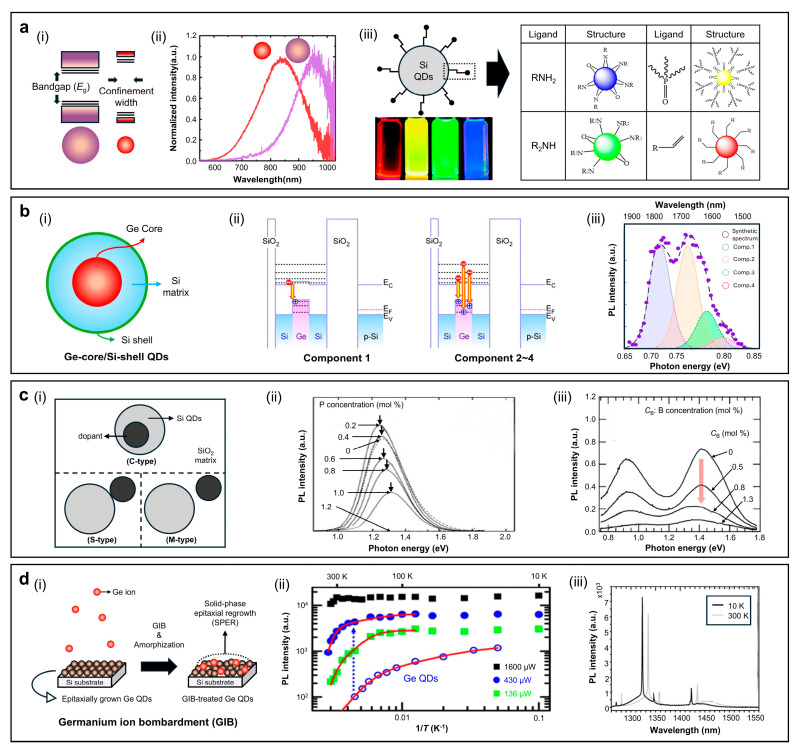
(**a**–**d**) Strategies for achieving efficient red/NIR emission in Si QD families by (**a**) quantum confinement effect: energy band diagrams illustrating size-dependent bandgaps in two QDs (**i**). Reproduced from [143], with permission from the Università degli Studi di Genova. Copyright (2018), corresponding PL spectra from the two different-sized QDs (**ii**). Reproduced from [144], with permission from the American Chemical Society. Copyright (2020), and surface ligand engineering: structure of Si QDs (**left**), functionalized with various ligands listed and digital images of 3–4 nm sized Si QDs with dodecyl-termination under an inert atmosphere (red), functionalized with trioctylphoshine (yellow), diphenylamine (green), and dodecylamine (blue) (**iii**). Reproduced from [145], with permission from the American Chemical Society. Copyright (2014). (**b**) The core/shell structure of Ge–core/Si–shell QDs: a schematic of type-II band alignment structure embedded in a Si matrix (**i**), energy band diagrams of radiative transitions (Comp. 1–4) (**ii**), and room-temperature PL spectrum deconvoluted into four Gaussian components, indicating strong hole confinement and efficient near-infrared emission (**iii**). Reproduced from [140], with permission under Creative Commons Attribution 4.0 (CC BY). Copyright (2023). (**c**) Doping strategies for Si QDs: a schematic classification into core (C-type), surface (S-type), and matrix (M-type) doping (**i**). Reproduced from [146], with permission from the Royal Society of Chemistry. Copyright (2016), PL intensity of boron-doped Si QDs showing a monotonic decrease with increasing dopant concentration (**ii**). Reproduced from [142], with permission from the American Physical Society. Copyright (2002), PL spectra of phosphorus-doped Si QDs exhibiting intensity variation and emission peak shift, depending on doping level (**iii**). Reproduced from [141], with permission from Springer Nature. Copyright (2000). (**d**) The Ge ion bombardment (GIB) strategy for Ge QDs: a schematic of the GIB process and subsequent solid-phase epitaxial regrowth (SPER), yielding partially amorphous/crystalline hybrid QDs (**i**), an Arrhenius plot of integrated PL intensity revealing negligible thermal quenching and a high activation energy (~350 meV) (**ii**), and PL spectra at 10 K and 300 K, demonstrating strong and stable emission from GIB-QDs, significantly superior to conventional crystalline Ge QDs (**iii**). Reproduced from [23], with permission from the American Chemical Society. Copyright (2016).

Red/NIR emissions in Si and Ge QDs can be systematically modulated through quantum confinement, surface passivation, and core–shell heterostructure engineering (Figure 8a,b). As shown in Figure 8a(i,ii), the quantum confinement governs bandgap tuning of QDs. The Si QDs fabricated from HSQ, for example, exhibit a PL redshift from 680 nm to 867 nm, when the size increases from 3.2 nm to 4.2 nm [78,79,80]. Similarly, the Si QDs (4.0 nm) grown on Si/SiO_2_ substrate by plasma-based method emit at 825 nm, with a PLQY of 90%, while those terminated with dodecyl ligands (4.5 nm) emit at 850 nm, with a PLQY of 67% [76,77]. By contrast, the Ge QDs grown by molecular beam epitaxy (MBE) show a PL redshift from 1340 nm to 1560 nm, when the size decreases from 10 nm to 4 nm. It indicates that quantum confinement and carrier delocalization decrease as the QD size increases [23]. Figure 8a(iii) illustrates the effect of surface ligands on the emission properties of Si QDs. Amine terminations (RNH_2_, R_2_NH) tend to create surface trap sites, which increase non-radiative recombination and cause a PL blueshift. In contrast, long-chain alkyl ligands (e.g., dodecyl or decyl) effectively passivate surface trap sites and stabilize the surface potential. For example, decyl-terminated Si QDs exhibit intense PL redshift and enhanced PLQY, reaching 77% [80]. Establishing core–shell heterostructures serves as a key design principle for group-IV QDs for efficient and stable NIR emission through band-offset engineering (Figure 8b). Ge–core/Si–shell QDs grown by reduced-pressure chemical vapor deposition (RPCVD) show type-II band alignment (Figure 8b(ii)). This configuration spatially separates carriers, with holes confined in the Ge core and electrons in the Si shell, which promotes indirect radiative recombination and a pronounced PL redshift. The PL spectrum in Figure 8b(iii) shows four Gaussian components (1–4) between 0.66 and 0.83 eV (1500–1870 nm), with a FWHM of ~84 nm, in alignment with quantized transitions originating from the type-II alignment [140].

#### 3.3.2. Doping

Doping provides a versatile means to tune the emission wavelength and radiative behavior of Si QDs. As shown in Figure 8c(i), dopants can be introduced into the QD system in different spatial configurations—within the core (C-type), on the surface (S-type), or in the surrounding matrix (M-type). Among these, C-type doping is most effective in generating electronically active states that influence radiative recombination, whereas S- and M-type doping primarily affects surface passivation and charge transport. For P-doped (n-type) Si QDs, PL intensity exhibits a non-monotonic dependence on dopant concentration (Figure 8c(ii)). At low concentrations (≤0.4 mol%), P atoms passivate interfacial dangling bonds, suppressing non-radiative recombination [141]. At higher concentrations (≥0.6 mol%), excess free electrons donated by substitutional P atoms induce Auger recombination, causing a slight PL blueshift, and eventually, PL quenching at 1.2 mol% [141,142]. Electron spin resonance (EPR) measurements further confirm that P doping in Si QDs is activated in ~5 nm Si QDs, since the hyperfine splitting is nearly twice compared to that of bulk Si:P due to strong quantum confinement [142]. In contrast, B-doped (p-type) Si QDs result in monotonic PL quenching (Figure 8c(iii)). Two emission bands (~0.85 eV and ~1.4 eV) appear, likely arising from slight size distribution within the QDs [141]. As the B concentration increases from 0 to 1.3 mol%, PL intensity at both peaks decreases, indicating that holes introduced by B acceptors enhance non-radiative, hole-assisted Auger recombination. Collectively, these results indicate that low-level n-type doping can enhance luminescence via defect passivation, whereas excessive donor or acceptor incorporation accelerates non-radiative recombination. Thus, both dopant species and their concentrations should be carefully controlled to achieve efficient red/NIR-emissive Si QDs.

#### 3.3.3. Ge Ion Bombardment (GIB)

Ge ion bombardment (GIB) is a technique that improves the optical performance of epitaxially as-grown Ge QDs by enhancing grain growth and strengthening carrier confinement. As illustrated in Figure 8d(i), surface amorphization of Ge QDs occurs by high-energy Ge^+^ irradiation during growth. Subsequent solid-phase epitaxial regrowth (SPER) recrystallizes the structure into a mixed crystalline–glassy configuration. This process suppresses non-radiative recombination by separating the original Ge/Si clusters into sub-4 nm crystalline Ge domains and amorphous Ge regions with a larger bandgap. As shown in Figure 8d(ii), the PL intensity of GIB-treated Ge QDs remains nearly constant from 10 to 300 K, with negligible thermal quenching. In contrast, conventional Ge QDs (blank circle plots in Figure 8d(ii)) without GIB treatment show complete PL quenching at room-temperature. The activation energy is calculated as ~350 meV by Arrhenius analysis, far exceeding the typical values of crystalline Ge QDs (60–80 meV). It confirms enhanced carrier confinement within the GIB-modified structure. The PL spectra in Figure 8d(iii) support the results above by showing a sharp emission peak near 1340 nm with an FWHM of ~70 nm at both 10 and 300 K. These results indicate that GIB-treated Ge QDs are promising for Si-compatible photonic and optoelectronic devices [23]. Table 5 summarizes composition, synthesis method, precursors, and several key optical properties of Si QDs and Ge QDs.

## 4. Applications of Red-Emissive, Cadmium-Free QDs

### 4.1. Carbon- or Graphene-Based QDs

#### 4.1.1. Light-Emitting Diodes (LEDs)

**Figure 9:**
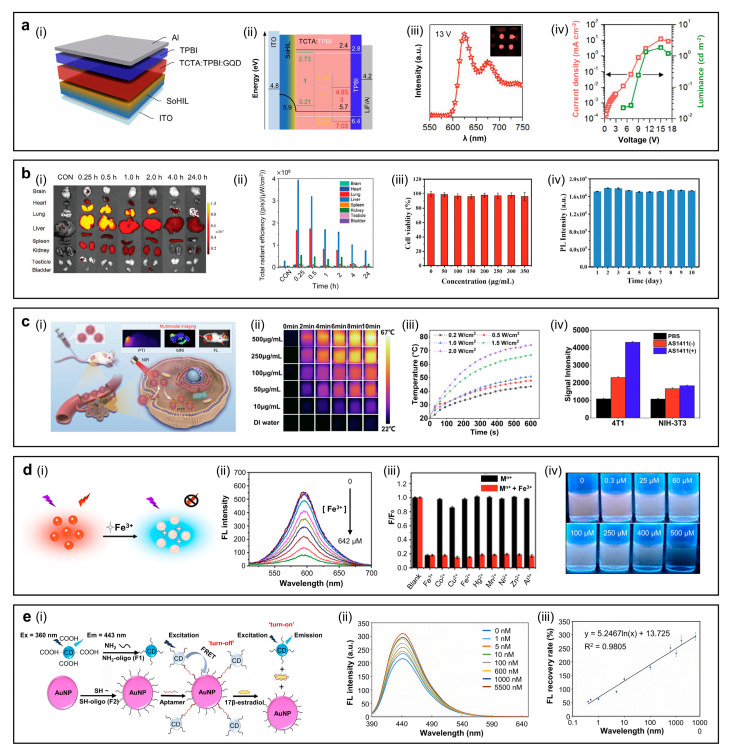
(**a**–**c**) Applications of red-emissive GQDs or CDs. (**a**) The device structure (**i**), energy levels (**ii**), electroluminescence (EL) spectra and digital image (inset) (**iii**), and current density and luminance curves (**iv**) of red-emitting GQD-LEDs. Reproduced from [60], with permission from Springer Nature. Copyright (2016). (**b**) Real-time ex vivo imaging of nude mice with intravenous injection of CDs at different time points (**i**), the biodistribution of CPDs after injection in nude mice at different time points, including semiquantitative FL intensity of brain, heart, lung, liver, spleen, kidney, testicle, and bladder (**ii**), viability of Hepa 1–6 cells at different concentrations of CDs in a 5% CO_2_ incubator at 37 °C for 24 h (**iii**), and the effect of different incubated time at ambient conditions on the fluorescence (FL) intensity of CDs (200 μg/mL) (**iv**). Reproduced from [67,151], with permission from Wiley–VCH. Copyright (2020), Elsevier. Copyright (2023). (**c**) A schematic illustration of the preparation of AS1411-gadolinium (Gd) doped red-emissive CDs and FL/magnetic resonance (MR)-guided photothermal therapy of the tumor (**i**), infrared thermal images of AS1411-Gd-CDs suspension of different concentrations exposed to NIR laser for 0–10 min (808 nm, 1 W cm^−1^) (**ii**), photothermal performance of AS1411-Gd-CDs (100 μg mL^−1^) exposed to NIR laser with different power densities (**iii**), and MR signal intensity of 4T1 and NIH3T3 cells incubated with PBS, Gd-CDs and AS1411-Gd-CDs, respectively (**iv**). Reproduced from [150], with permission from Elsevier. Copyright (2022). (**d**) The FL sensing mechanism for the detection of Fe^3+^ (**i**), FL spectra of B, N, S-*co*-doped CDs (BNS-CDs) upon the addition of various concentrations Fe^3+^ (**ii**), FL intensity ratio (F/F_0_) of the BNS-CDs in the presence of various metal ions (**iii**), and photographs of BNS-CDs upon the addition of different concentrations of Fe^3+^ (**iv**). Reproduced from [152], with permission from the American Chemical Society. Copyright (2017). (**e**) A schematic illustration of the FRET aptasensor for 17β-estradiol detection using CDs (**i**), FL spectra (**ii**), and FL intensity recovery efficiency (**iii**) under different concentrations of 17β-estradiol. Reproduced from [153], with permission from the Royal Society of Chemistry. Copyright (2023).

Due to their interesting optical properties, CDs or GQDs have been applied in diverse fields, including LEDs [51,147,148,149], bioimaging, multicolor emission of R-GQDs in various solvents, and photothermal therapy (PTT). The LED device incorporates a self-organized polymeric hole-injection layer (SOHIL), whose work function rises monotonically from 5.2 eV at the bottom interface to 5.95 eV at the top surface; this built-in gradient lowers the hole–injection barrier and enables efficient hole transfer into the TCTA:TPBI:GQD emissive layer (Figure 9a(i,ii)) [60]. Under applied bias, electrons are injected through the Al/LiF/TPBI cathode stack, while holes are delivered via the ITO/SOHIL anode. Figure 9a(iii) shows the electroluminescence (EL) spectrum peaking at *λ* = 620 nm with an FWHM of ~50 nm, demonstrating relatively narrow red emission suitable for display applications. The current density–voltage–luminance (J-V-L) characteristics reveal a maximum luminance (*L*_max_) of ~2 cd m^−2^ and an external quantum efficiency (EQE) of ~0.1% (Figure 9a(iv)). These results confirm the proof-of-concept operation, but highlight that there is significant room to improve device performance through charge balance and solid-state PLQYs [52].

#### 4.1.2. Bioimaging

Deep-red emissive CDs with narrow FWHM are highly promising for deep-tissue bioimaging. Figure 9b(i) shows real-time ex vivo FL images of organs collected 0.25–24 h after intravenous administration of CDs to nude mice [67]. The highest initial signal is observed in the liver and lung, and decreases rapidly to a minimum after 24 h. Semiquantitative analysis of total radiant efficiency supports these results, that the CDs accumulate mainly in the liver and lung within 0.25–1 h, then decline rapidly. The other organs show much lower uptake throughout the study period (Figure 9b(ii)). The principal advantage of CDs or GQDs lies in their low cytotoxicity, which distinguishes them from those of inorganic QDs that may exhibit potential toxicity associated with In-core materials or ZnS shells. Cell viability tests have been performed using Hepa 1–6 cells treated with 0–350 µg mL^−1^ CDs for 24 h at 37 °C. As shown in Figure 9b(ii), the cell viability remains above 90% across this concentration range, confirming very low cytotoxicity of the CDs. Figure 9b(iii) displays PL spectra of a 200 µg mL^−1^ CD solution stored at room temperature for 10 days. The PL intensity remains nearly constant, indicating excellent photostability and supporting its suitability for bioimaging.

#### 4.1.3. Photothermal Therapy (PTT)

The AS1411-Gd-CDs nanoprobes enable integrated tumor-targeted delivery, tri-modal imaging (photoacoustic tomography (PAT), magnetic resonance imaging (MRI), fluorescence (FL)), and 808 nm laser-triggered photothermal therapy through selective aptamer–nucleolin recognition (Figure 9c(i)) [150]. The IR images in Figure 9c(ii) show a clear color progression from 22 °C to 67 °C during 10 min of 808 nm laser irradiation. The heating efficiency increases with the concentration of nanoprobe, while remaining nearly unchanged in pure water, confirming effective photothermal conversion. Figure 9c(iii) indicates the steady-state temperature change at different laser powers over time. At a fixed concentration of 100 µg mL^−1^, steady-state temperatures increase from ~44 °C at 0.2 W cm^−2^ to ~74 °C at 2.0 W cm^−2^ after 600 s of laser irradiation, demonstrating strong laser power-dependent photothermal performance. In T_1_-weighted MR images of 4T1 tumor cells, AS1411-targeted Gd-CDs showed more than twofold signal enhancement compared to non-targeted Gd-CDs, whereas NIH-3T3 normal cells exhibited only minimal enhancement (Figure 9c(iv)). This confirms selective tumor cell uptake and high relaxivity for targeted MRI contrast.

#### 4.1.4. Sensing

B, N, S co-doped CDs (BNS-CDs) function as a FL nanosensor for the detection of Fe^3+^ via FL quenching (Figure 9d(i)) [152]. The BNS-CDs are synthesized by one-step hydrothermal treatment in 2,5-diaminobenzenesulfonic acid and 4-aminophenylboronic acid hydrochloride, exhibiting red emission centered at 595 nm at various excitation wavelengths of 365–500 nm. Heteroatom doping (B, N, S) modulates the bandgap by altering electron density, which increases surface defects and reduces non-radiative recombination. As shown in Figure 9d(ii), the FL intensity at 595 nm decreases progressively with increasing concentrations of Fe^3+^ (0–642 μM). The nanosensor demonstrates high sensitivity for Fe^3+^ in the range of 0.3–546 μM, with a detection limit of 90 nM, along with good selectivity toward Fe^3+^ over other metal ions (Figure 9d(iii)). The FL quenching behavior is further confirmed by FL images, which show that the red FL was gradually diminished with increasing Fe^3+^ concentrations (Figure 9d(iv)).

Another study reported a “turn-on” FRET aptasensor based on CDs and gold nanoparticles (Au NPs) for the detection of 17β-estradiol [153]. Excess 17β-estradiol can disturb the endocrine system, which emphasizes the importance of sensitive detection. The sensing mechanism of 17β-estradiol detection by the FRET aptasensor is illustrated in Figure 9e(i). The CDs act as FL donors, while the AuNPs serve as quenchers. Amino-modified oligos (F1) are grafted onto the surface of CDs via carbodiimide coupling, and thiol-modified oligos (F2) are conjugated to the Au NPs through strong Au-S covalent bonds. Then, the CDs-F1-aptamer-F2-Au NPs assembly is formed by co-hybridizing F1 and F2 with the 17β-estradiol aptamer. This brings the CDs and the Au NPs into close proximity (within 10 nm). Under 360 nm excitation, the FL energy of CDs is transferred to the AuNPs, thereby enabling the FRET that is characterized by the decrease in FL intensity (“turn-off”). In the presence of 17β-estradiol, the aptamer specifically binds to the 17β-estradiol, and the assemblies are dissociated. The CDs and the AuNPs are then spatially separated in the solution. As a result, the FRET is suppressed, and the FL of CDs is recovered (“turn-on”) upon excitation. Upon the increase in the 17β-estradiol concentrations, the FL intensity of the CDs increases (Figure 9e(ii)), exhibiting a good linear relationship between FL recovery efficiency and the concentration of 17β-estradiol (Figure 9e(iii)). Based on this calibration, the limit of detection (LOD) is determined to be as low as 245 pM. Sensing performance of FL-based sensors using CDs or GQDs is summarized in Table 6.

### 4.2. Inorganic Core–Shell QDs

#### 4.2.1. Light-Emitting Diodes (LEDs)

##### Indium Phosphide (InP)

**Figure 10:**
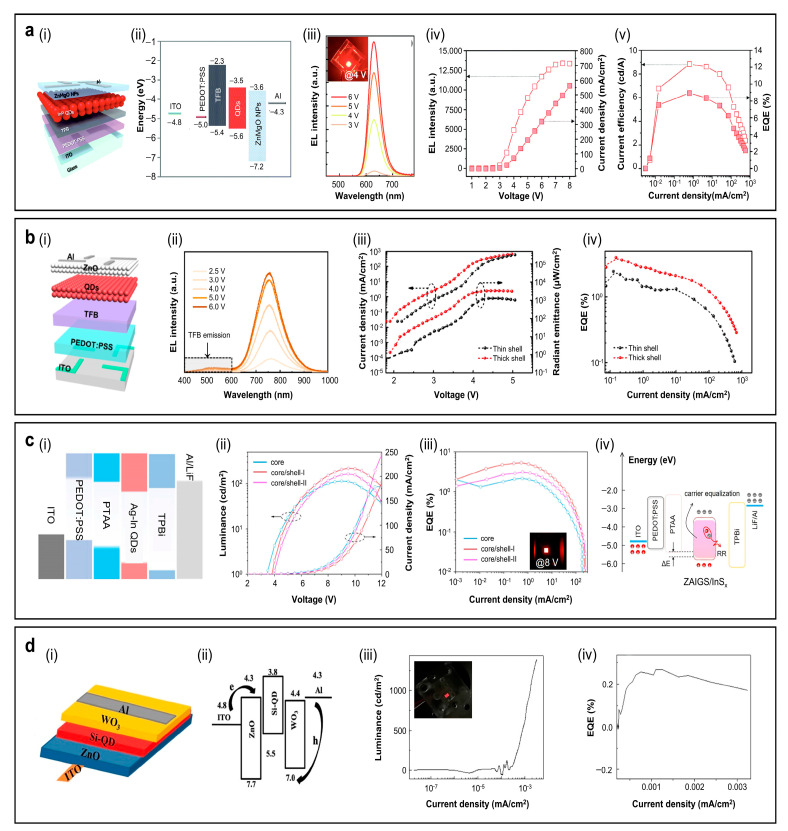
QLEDs applications of red/NIR-emissive, Cd-free inorganic QDs. (**a**) The device structure (**i**), energy levels (**ii**), electroluminescence (EL) spectra and digital image (inset) (**iii**), EL intensity and current density curves (**iv**), and current efficiency and EQE curves (**v**) of a bottom-emitting red QLEDs employing InP/thick-ZnSe/ZnS core/shell QDs as the emitting layer (EML). Reproduced from [68], with permission from the Royal Society of Chemistry. Copyright (2022). (**b**) The device structure (**i**), EL spectra at various voltages (**ii**), current density–voltage (J-V) and radiant emittance curves (**iii**), and EQE characteristics (**iv**) of NIR-emitting QLEDs based on thin-shell and thick-shell Zn:CuInSe_2_ core/shell QDs. Reproduced from [18], with permission from Optica Publishing Group. Copyright (2022). (**c**) The device structure (**i**), current density–voltage–luminescence (J-V-L) curves (**ii**), EQE curves (**iii**) of red QLEDs based on ZAIGS core QDs and ZAIGS/InS_x_ core/shell-I, core/shell- II QDs, and (**iv**) energy diagram of carrier distribution and recombination in ZAIGS/InS_x_-based QLEDs. Reproduced from [124], with permission from the American Chemical Society. Copyright (2023). (**d**) An inverted device structure (**i**), the corresponding flat band diagram (**ii**), current density-luminance (L-V) curve and digital image (inset) (**iii**), and EQE curve (**iv**) of red Si-QLEDs. Reproduced from [157], with permission from MDPI. Copyright (2019).

Figure 10a illustrates the device structure of bottom-emitting red QLEDs, employing InP/thick-ZnSe/ZnS core/shell QDs as the emitting layer (EML) [68]. The device adopts a typical multilayer configuration consisting of an ITO anode, a PEDOT:PSS hole-injection layer (HIL), a TFB hole-transport layer (HTL), a QD emitting layer (EML), a ZnMgO nanoparticle-based electron-transport layer (ETL), and an Al cathode, all fabricated via solution-processed methods (Figure 10a(i)). The corresponding energy-band diagram (Figure 10a(ii)) shows that the valence band maximum (VBM) and conduction band minimum (CBM) of the InP/thick-ZnSe/ZnS QDs are –5.6 eV and –3.5 eV, respectively, as determined by absorption spectrum and ultraviolet photoelectron spectroscopy (UPS) measurements. This careful band alignment serves an important role in the efficient and balanced injection of electrons and holes. The device exhibits excellent spectral stability (Figure 10a(iii)) and high performance, achieving a maximum luminance of 13,395 cd m^−2^ at a current density of 496 mA cm^−2^ under 7.5 V (Figure 10a(iv)), which is significantly higher than that of aminophosphine-derived InP QD-based red QLEDs (1400–2849 cd m^−2^). Furthermore, a maximum current efficiency of 8.8 cd A^−1^ and an EQE of 8.9% were obtained at a low current density of 0.6 mA cm^−2^, with high efficiency maintained at practical luminance levels (Figure 10a(v)).

Previous studies on red-emissive InP QDs for QLEDs have demonstrated notable performance improvements in both bottom- and top-emitting structures. For example, in the top-emitting device utilizing the microcavity effect, the FWHM was reduced from 54 nm to 32 nm, resulting in high color purity red emission comparable to that of a CdSe QD-based device [156]. In addition, inverted QLEDs incorporating InP/ZnSe/ZnS core/shell QDs achieved red EL at 607 nm with a maximum luminance, a peak current efficiency, and EQE of ~1600 cd m^−2^, 13.6 cd A^−1^, and 6.6%, respectively. This is attributed to balanced charge recombination and reduced non-radiative losses by the thick ZnS shell [156].

##### Copper–Indium (Cu-In) Chalcogenides

NIR-I QLEDs have been demonstrated using Zn:CuInSe_2_ QDs as the EML (Figure 10b) [18]. As shown in Figure 10b(i), the device comprises an ITO anode, a PEDOT:PSS HIL, a TFB HTL, a Zn:CuInSe_2_ QD EML, a ZnO ETL, and an Al cathode; all layers are deposited via solution-processing. This device configuration is designed to optimize charge balance in I–III–VI QLEDs, which typically exhibit hole-dominant injection characteristics. The EL spectra recorded at applied voltages ranging from 2.5 to 6.0 V show stable NIR-I emission, with a dominant peak in the 700–750 nm range. A weak TFB-related emission (~500 nm) appears at low bias and rapidly diminishes at higher voltages, indicating that radiative recombination occurs predominantly within the QD layer. Figure 10b(iii) compares the J–V characteristics and radiant emittance of devices based on thin-shell Zn:CuInSe_2_/ZnS QDs and thick-shell Zn:CuInSe_2_/ZnS//ZnS QDs. The devices incorporating thick shells exhibit markedly higher current density and radiant emittance across the entire voltage range, with a maximum radiant emittance increase exceeding 160% relative to thin shell counterparts. This improvement is attributed to enhanced surface passivation by the thick outer ZnS shell, which suppresses nonradiative recombination and enhances charge-injection balance [18,41,115,120]. Correspondingly, EQE-current density characteristics reveal a rapid efficiency roll-off in thin-shell devices, whereas thick-shell devices maintain substantially higher EQE over a wide current density range (Figure 10b(iv)). The maximum EQE of thick-shell devices is approximately 60% higher than that of their thin-shell counterparts. This enhancement is ascribed to the dual ZnS shell structure, which effectively mitigates charge-injection asymmetry and promotes more balanced electron–hole recombination within the EML [18].

##### Silver–Indium (Ag-In) Chalcogenides

Figure 10c shows the structure of QLEDs employing the Ag–In QDs in the EML. Specifically, these QDs include core Zn-Ag-In-Ga-S (ZAIGS)-only QDs, ZAIGS/In-rich 2-layer InS_x_ shell (core/shell-I), and ZAIGS/S-rich 3-layer InS_x_ shell (core/shell-II) (labeled simply as “Ag-In QDs” in Figure 10c(i)) [124]. Luminance and EQE increased from 114.4 cd·m^−2^ and 2.15% (core) to 218.1 cd·m^−2^ and 5.32% (core/shell-I) (Figure 10c(ii,iii)), consistent with PLQY enhancement from 19.8% at core to 59.4% at core/shell-II. An improved surface planarity suppresses electrical leakage and thus enhances emission efficiency. Mechanistically, coating of the InS_x_ shell effectively reduced the electron trap-filled limit voltage (*V*_TFL_) and increased the hole *V*_TFL_. This is attributed to the shift in the valence band (VB) to deeper energy levels due to the passivation of S vacancy-induced electron traps. These changes led to more balanced charge injection and transport, directly contributing to the enhanced efficiency and operational stability of QLEDs based on ZAIGS/InS_x_ QDs (Figure 10c(iv)).

##### Silicon or Germanium (Si or Ge)

Red/NIR-emissive Si QDs represent a low-toxicity, thermally robust alternative to conventional Cd-based QDs. Figure 10d(i) shows an all-inorganic Si QD QLED with an inverted architecture of ITO/ZnO/Si QDs/WO_3_/Al. In this structure, a ZnO nanoparticle layer (~22 nm) functions as the electron injection and transport layer, while a WO_3_ nanoparticle layer (~13 nm) serves as the hole injection and transport layer, sandwiching a spin-coated emissive layer of decane-terminated Si QDs (Figure 10d(ii)). All inorganic layers are deposited via solution-based spin-coating using orthogonal solvents, enabling damage-free multilayer fabrication and enhanced device robustness. Under electrical bias, the device exhibits bright NIR-I EL (~729 nm) originating from radiative recombination within the Si QD layer, as confirmed by the digital photograph inset in Figure 10d(iii). The luminance–current density characteristics show a turn-on at ~3 V, followed by a rapid luminance increase beyond ~10^−4^–10^−3^ A cm^−2^, reaching ~1400 cd m^−2^ at ~3.2 × 10^−3^ A cm^−2^. This performance demonstrates efficient charge injection and high-brightness operation from solution-processed Si QDs despite silicon’s indirect bandgap nature. The EQE curve shows a peak EQE of ~0.25% at low current densities (~1.0–1.3 × 10^−3^ A cm^−2^), followed by mild roll-off at higher currents (Figure 10d(iv)). Although lower than that of hybrid Si-QLEDs, the same QDs exhibit a thin-film PLQY of ~18%, indicating that the EQE is primarily limited by device architecture rather than emissive efficiency. In particular, non-ideal band alignment and a large hole–injection barrier at the Si QD/WO_3_ interface hinder efficient hole injection and reduce EL efficiency. Nevertheless, the modest EQE roll-off and stable emission highlight the intrinsic advantages of the fully inorganic architecture, including superior operational stability and humidity resistance compared to organic-inorganic hybrid QLEDs. These results underscore the potential of Si QD-based all-inorganic QLEDs for stable, cadmium-free optoelectronic applications [157].

#### 4.2.2. Bioimaging

##### Indium Phosphide (InP)

**Figure 11:**
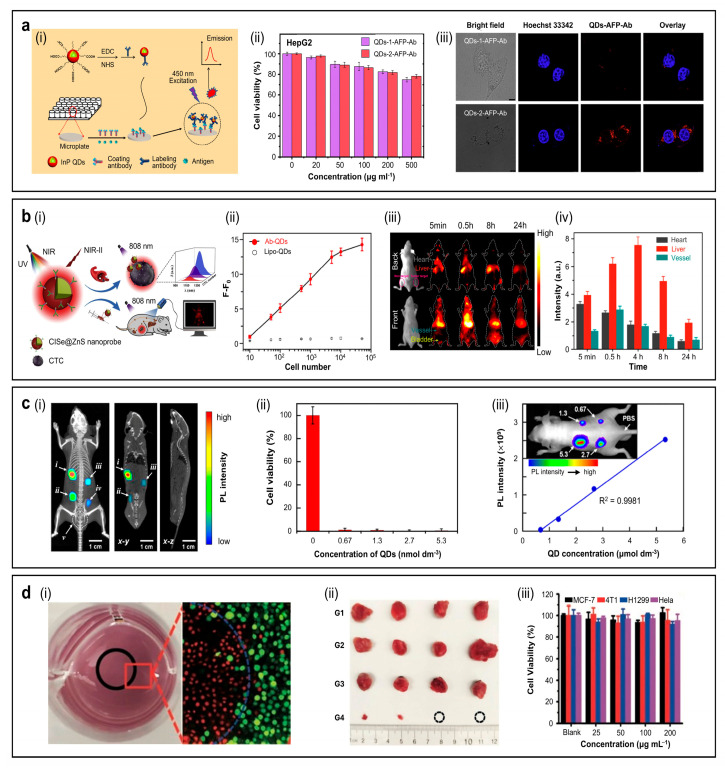
Bioimaging applications of red/NIR-emissive, Cd-free inorganic QDs. (**a**) a schematic illustration of the QD-based fluorescent-linked immunosorbent assay (QDs-FLISA), using InP QDs (**i**), in vitro cell viability of HepG2 cells treated with varying concentrations of QDs-1-AFP-Ab and QDs-2-AFP-Ab probes (**ii**), and in vitro imaging of cancer cells by QDs-1-AFP-Ab and QDs-2-AFP-Ab probes (**iii**). Hoechst 33342 was used to counterstain the nuclei with blue. Reproduced from [74], with permission from Wiley–VCH. Copyright (2022). (**b**) A schematic illustration of broadband-excitable CISe@ZnS nanoprobes for CTC detection and NIR-II bioimaging (**i**), calibration curve showing NIR-II PL response as a function of MCF-7 cell number for Ab-QDs and Lipo-QDs (**ii**), in vivo NIR-II fluorescence images of tumor-bearing mice from back and front views at different time points post-injection of Ab-QDs (**iii**), and quantitative NIR-II PL intensities in the heart, liver, and vessel regions over time (**iv**). Reproduced from [117], with permission from Elsevier. Copyright (2020). (**c**) A three-dimensional PL image overlaid and X-ray CT scan of a mouse subcutaneously injected in the dorsal region with DSPC-AIGSe@GaS_x_ liposome dispersions (50 mm^3^ each) (**i**), cell viability of A431 cells as a function of DSPC–AIGSe@GaSₓ liposome concentration (**ii**), and PL spectra detected through the skin of a mouse and QD concentration in DSPC-AIGSe@GaS_x_ liposome dispersions and inset showing in vivo PL images of mice subcutaneously injected in the dorsal region with DSPC–AIGSe@GaSₓ liposome dispersions (50 mm^3^ each) (**iii**). Reproduced from [136], with permission from the American Chemical Society. Copyright (2020). (**d**) Live/dead fluorescence imaging of 4T1 cancer cells after photothermal treatment, showing extensive cell death (red, PI) within the NIR laser spot and intact live cells (green, calcein-AM) outside the irradiated region (**i**), digital images of mice tumors after different treatments (G1: Control; G2: NIR; G3: PEG@Ge QDs; G4: PEG@Ge QDs+NIR (808 nm, 2 W cm^−2^) (**ii**), and cell viability of multiple cell lines (MCF-7, 4T1, H1299, HeLa) treated with increasing concentrations of PEG@Ge QDs (**iii**). Reproduced from [158], with permission from Wiley–VCH. Copyright (2019).

The QD-based fluorescent-linked immunosorbent assay (QDs-FLISA) utilizes covalent bonding between the –COOH groups on the QD surface and the –NH_2_ groups of antibodies, which is achieved through EDC/sulfo-NHS chemistry to form QDs–alpha–fetoprotein (AFP)-antibody (Ab) fluorescent probes (Figure 11a(i)). These probes enable FL detection through a sandwich immunoassay. A capture antibody immobilized on a microplate binds to the AFP antigens, and a detection antibody conjugated to the QDs completes the complex. A quantitative measurement of FL was performed using 450 nm excitation. Biocompatibility has been evaluated using (3-(4,5-dimethyl-2-thiazol)-2,5-diphenyltetrazolium bromide) (MTT) assays on cells treated with varying concentrations of QDs-1-AFP-Ab and QDs-2-AFP-Ab probes. Water-soluble InP QDs, prepared via simple ligand exchange and those obtained by a photochemical process, are designated as QDs-1 and QDs-2, respectively. Both probes show over 60% cell viability even at a high concentration of 500 μg mL^−1^, indicating negligible cytotoxicity (Figure 11a(ii)). This result suggests that InP QDs possess excellent biocompatibility and can serve as safe fluorescent probes for biological applications, including bioimaging. To further evaluate target-specific binding affinity toward hepatocellular carcinoma (HCC) cells, HepG2 cells are treated with the two probes for 6 h and imaged using fluorescence and confocal laser scanning microscopy (CLSM). The FL signal of QDs-1-AFP-Ab is weak, with slight aggregation around the nucleus, while that of QDs-2-AFP-Ab is strong within the cytoplasm. This difference is attributed to the lower density of surface ligands on QDs-1, which limits antibody binding and reduces colloidal stability in aqueous media [74].

##### Copper–Indium (Cu-In) Chalcogenides

The CISe@ZnS-based NIR-II biosensing assay employs broadband-excitable luminescent nanoprobes for sensitive detection of circulating tumor cells (CTCs) (Figure 11b(i)) [117]. Monodisperse CISe QDs have been synthesized via the hot-injection method. Controlling the stoichiometric ratio of Se/In enables the precise tuning of emission wavelengths in the range of 920–1224 nm. A thin ZnS shell formed by epitaxial growth effectively passivates surface defects and suppresses nonradiative recombination. This prevents Cu^+^ oxidation, yielding a high PLQY of 21.8% in the NIR-II window. Targeted probes are produced through covalent conjugation of QDs with anti-EpCAM antibodies. These probes are capable of selectively recognizing EpCAM-positive MCF-7 breast cancer cells. The fluorescence signal increases monotonically with cell number, enabling quantitative detection down to 12 cells per well in a 96-well plate (Figure 11b(ii)). Biocompatibility and in vivo behavior are evaluated after injecting antibody-conjugated CISe@ZnS nanoprobes into the tail vein of mice with tumors. NIR-II fluorescence imaging reveals rapid systemic circulation and initial accumulation in the heart and major vessels within minutes, followed by progressive uptake in the liver and tumor tissues over 0.5–8 h of post-injection (Figure 11b(iii)). A temporal biodistribution trend is confirmed by quantitative analysis of FL intensity in the heart, liver, and vessels (Figure 11b(iv)), which is consistent with ICP-AES results showing Cu concentrations measured in these organs. Tumor-targeted accumulation gradually increases with time, resulting in clearer visualization of tumors. Whilst the transient bladder signals indicate an efficient renal clearance. These results demonstrate that CISe@ZnS nanoprobes combine high biocompatibility, strong tumor-targeting specificity, and favorable in vivo clearance, highlighting their potential for ultrasensitive CTC detection and tumor-targeted NIR-II bioimaging.

##### Silver–Indium (Ag-In) Chalcogenides

In vivo 3D PL imaging of 1,2-distearoyl-*sn*-glycero-3-phosphocholine (DSPC)-AIGSe@GaS_x_ liposomes exhibits strong NIR-I emission, enabling real-time tracking in mice up to 5 mm beneath the skin (Figure 11c(i)). This demonstrates the excellent tissue-penetration capability and stability of the AIGSe@GaSₓ QDs under physiological conditions. Encapsulation of AIGSe@GaSₓ QDs within the DSPC lipid bilayer not only preserves their emission efficiency but also provides a biocompatible carrier for deep-tissue optical imaging [136]. Cytotoxicity studies with A431 cells have shown severe toxicity across all concentrations, with cell viability dropping below 2% within 1 h (Figure 11c(ii)). This high toxicity is likely attributed to unpassivated or reactive surface ligands, such as residual amines or metal ions exposed on the QD surface, rather than the lipid carrier. These findings highlight the necessity of ligand exchange or additional surface passivation to ensure biocompatibility for potential in vivo applications. Furthermore, PL intensity shows a linear dependence on QD concentration (Figure 11c(iii)), suggesting their potential use for quantitative in vivo bioimaging. Such proportional dependence indicates the feasibility of using these QDs for quantitative bioimaging or real-time biodistribution tracking, provided cytotoxicity issues are properly addressed [136].

##### Silicon or Germanium (Si or Ge)

Figure 11d(i) shows the results of FL imaging for live/dead 4T1 cells exposed to localized NIR laser irradiation. A distinct boundary is observed between the laser-irradiated and the non-irradiated regions. The cells within the laser-irradiated region undergo extensive photothermal ablation and exhibit a strong PI (red) FL signal. However, the surrounding cells remain alive and display a calcein-AM FL signal (green). Digital photographs in Figure 11d(ii) show excised tumors after different treatments (G1: control, G2: NIR only, G3: Ge QDs only, G4: Ge QDs + NIR). While the tumor sizes in G1–G3 are similar, the G4 group shows almost complete removal of the tumor mass. This confirms the potent in vivo photothermal efficacy of Ge QDs under NIR irradiation. Figure 11d(iii) summarizes the cytocompatibility of Ge QDs across several cell lines (MCF-7, 4T1, H1299, and HeLa). All groups maintain over 90% at concentrations up to 200 μg mL^−1^, which demonstrates excellent biocompatibility. Overall, these results establish Ge QDs as promising agents not only for effective NIR photothermal therapy but also for broader applications in FL-based bioimaging [158].

#### 4.2.3. Sensing

##### Indium Phosphide (InP)

**Figure 12:**
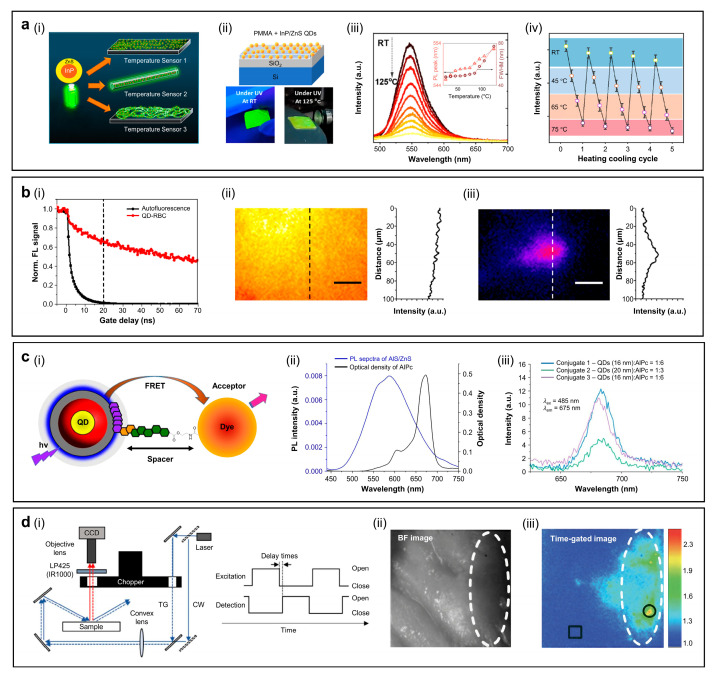
Sensor applications of red/NIR-emissive, Cd-free inorganic QDs. (**a**) A schematic illustration of the InP/ZnS QD-based temperature sensors (**i**), a scheme of the PMMA+InP/ZnS thin film temperature sensor and photographs of the sensor under UV light at room-temperature and at 125 °C (**ii**), temperature-dependent PL spectra (the inset shows the variation of the PL peak and FWHM with temperature) (**iii**), and heating−cooling cyclic measurement (**iv**) of PMMA+InP/ZnS QDs. Reproduced from [159], with permission from the American Chemical Society. Copyright (2024). (**b**) Normalized FL spectra of QD-loaded red blood cells (RBCs) as a function of gate delay (gate duration: 130 ns) (**i**), and in vivo FL images with intensity profiles along the dotted line at delay = 0 (**ii**), and 20 ns (**iii**), showing a circulating QD-labeled RBC (scale bar = 40 μm). Reproduced from [160], with permission from the American Chemical Society. Copyright (2019). (**c**) A schematic illustration of the QD-based FRET sensor (**i**), absorption and PL spectra of aluminum phthalocyanine-arginine (AlPc), and AIS/ZnS QDs, respectively (**ii**), and PL spectra of AIS/ZnS QDs-AlPc conjugates (**iii**). Reproduced from [160,162], with permission from the American Chemical Society and MDPI. Copyright (2020 and 2022). (**d**) The experimental setup and PL excitation/detection timing chart for time-gated FL imaging in the NIR-II region using Si QDs (**i**), the bright field (BF) (**ii**), and time-gated (**iii**) images of a chicken wing. A solution of Si QDs was injected into the region surrounded by a dashed circle. Reproduced from [161], with permission from the Royal Society of Chemistry. Copyright (2018).

QD-based temperature sensing platforms that exploit reversible FL signal changes with temperature variation are promising due to the high PLQYs and narrow FWHM of QDs. As temperature increases, exciton–phonon interactions become more pronounced, promoting non-radiative relaxation pathways. This reduces radiative recombination efficiency and leads to thermal quenching. Three different architectures of InP/ZnS QD-based temperature sensors employing poly(methyl methacrylate) (PMMA) as the host matrix have been proposed [159], consisting of a planar InP/ZnS QDs/PMMA thin film deposited on a Si wafer, InP/ZnS QDs/PMMA-filled borosilicate fibers, and InP/ZnS QDs-doped electrospun PMMA nanofibers deposited on a Si wafer (Figure 12a(i)). Figure 12a(ii) shows a schematic of the planar device and FL images under UV irradiation at 25 °C and 125 °C, respectively. Surface cracks in PMMA expose QDs to the atmosphere upon heating, which causes photooxidation of QDs and promotes non-radiative recombination. The PL spectra in Figure 12a(iii) indicate that the PL intensity gradually decreases with increasing temperature from 25 °C to 125 °C. A redshift of 0.1 nm/°C in the peak position (from 544 to 554 nm) and a broadening of the FWHM (from 43 to 80 nm) are also observed. While the FL response is reversible over four heating-cooling cycles between 25 and 75 °C, the sensor lost its reversibility above 75 °C (Figure 12a(iv)).

##### Copper–Indium (Cu-In) Chalcogenides

Time-gated imaging of NIR-I emitting (~800 nm) ZnCuInSe/ZnS QDs with FL lifetimes in the range of 150–300 ns enables efficient rejection of fast autofluorescence and the selective detection of QD emission, thereby significantly enhancing sensitivity [159]. The QDs exhibit long FL lifetimes due to hole trapping and electron delocalization, leading to reduced electron–hole overlap and slower recombination. They are solubilized in water using a multidentate imidazole−zwitterionic block copolymer and subsequently used to label model erythrocytes. Figure 12b(i) shows the temporal evolution of autofluorescence as a function of the delay between pulsed excitation and gate opening. The detected autofluorescence decays rapidly with characteristic lifetimes of 1–5 ns, while that of the detected FL of QD-labeled RBC remains a strong signal, even at long gate delays, with lifetimes of 100–200 ns. Figure 12b(ii) indicates that without a delay, all autofluorescence photons are detected by the charge-coupled camera (CCD). With a 20 ns delay, autofluorescence is reduced to the level of the instrumental noise, as confirmed by the intensity profiles (Figure 12b(iii)). This enables efficient detection of QD-labeled RBC, achieving ~99% autofluorescence rejection while preserving >60% of the QD FL signal.

##### Silver–Indium (Ag-In) Chalcogenides

Inorganic QDs are advantageous for FRET sensors. A large effective Stokes’ shift improves the signal-to-noise ratio (SNR), and tunability of size and composition allows for precise control of emission properties. Figure 12c(i) schematically illustrates the working principle of the QD-based FRET system, where the QDs act as donors and dye molecules as acceptors. Upon excitation, generated excitons in QDs can non-radiatively transfer their energy to nearby acceptors. The distance between the donor and the acceptor is a critical parameter in determining the FRET efficiency, which is typically controlled within a few nanometers using spacer molecules, such as surface linkers or biomolecules. The degree of spectral overlap between the emission of the donor and the absorption of the acceptor is also important. Figure 12c(ii) shows spectral overlap between the emission of AIS/ZnS QDs and the optical density (absorption) of aluminum phthalocyanine–arginine (AlPc) [160]. The PL spectra in Figure 12c(iii) show that conjugates 1–3 exhibit the AlPc emission at 675 nm upon excitation at 485 nm. It clearly indicates that a photon energy is transferred from the QDs to the AlPc through FRET, since the absorption of that energy does not occur in AlPc.

##### Silicon or Germanium (Si or Ge)

Compared to the conventional NIR-I window (700–950 nm), the NIR-II window (1000–1700 nm) exhibits reduced tissue scattering and autofluorescence, which allows for deep-tissue penetration at centimeter-scale with high spatial resolution. Si is an indirect bandgap material that shows an intrinsically slow carrier relaxation rate (tens to hundreds of μm), making Si QDs with an extended emission range to the NIR-II region highly promising. A possible strategy to achieve NIR-II emission is the introduction of in-gap states (donor and acceptor) via B and P co-doping [161], which shifts the emission energy by several hundred meV relative to intrinsic Si QDs of comparable size. Figure 12d(i) shows the measurement setup for time-gated FL imaging, using a mechanical chopper and a CCD camera [161]. For demonstration, a colloidal solution of B, P co-doped Si QDs (0.12 mg ml^−1^) is injected into a chicken wing sample. The bright-field image acquired prior to laser excitation serves as an anatomical reference for the subsequent FL imaging experiments. The dashed circle indicates the subcutaneous injection site, which defines the expected location of the FL signal (Figure 12d(ii)). Figure 12d(iii) displays the corresponding time-gated image of the same region, where the IR1000 long-pass filter is removed, and thus all emission above 425 nm is collected. Owing to the long FL lifetime (~200 μs) of the co-doped Si QDs, a high-contrast image is obtained even without the filter. The signal-to-background ratio is 3.9 for the time-gated imaging, which can be improved by decreasing the delay time [161]. Sensing performance of FL-based sensors using InP QDs, Cu/Ag-In chalcogenide QDs, and Si QDs is summarized in Table 7.

##### Design Rules for Sensing Applications

In sensor applications, design rules should be carefully tailored, considering the target analytes, the operating environment, and the optical detection method. In terms of the emission pathways of QDs, excitonic (inter-band) emission is ideal for multiplex detection as it offers high molar absorptivity and narrow FWHM characteristics. On the other hand, the emission originating from surface defects is useful for the FL “turn-on/off” single detection as it can minimize scattering interference due to the large Stokes’ shift. Meanwhile, several failure modes in detection may occur when the sensing media is biological fluid, such as sweat, interstitial fluid, or blood. These and other failure modes typically accompany degradation of QDs, which includes aggregation due to high salinity or pH drift, non-specific adsorption of biomolecules on the QD surface, or photooxidation, all of which adversely affect signal-to-noise ratio (SNR). To overcome these problems, precise control of the surface-to-volume ratio and the improvement of the charge transfer (CT) or FRET efficiency between the QDs and the analytes can enhance the sensitivity and lower the LOD. Selectivity can be improved through grafting antifouling ligands (e.g., poly(ethylene glycol), zwitterionic polymers) or through specific functionalization of aptamers or antibodies by preventing non-specific adsorption. Encapsulation of the QDs in sensor applications implies another strategy to improve the stability of wearable sensors. In this case, the QDs can be incorporated in polymer matrices or hydrophobic shells to protect them from sweat-induced pH drift and oxygen-induced photobleaching.

**Table 7:** Summary of FL sensors, using InP QDs, Cu/Ag-In chalcogenide QDs, and Si QDs.

**Sensing** **mechanism**	**QD type**	**Target analyte**	**LOD**	**Linear range**	**Year**	**Ref.**
FL intensity turn-on	InP/ZnS	CH_3_OH vapor	NA	NA	2026	[163]
FL intensity turn-off	InP/ZnS	Temperature	NA	25–120 °C	2024	[159]
FL intensity turn-off	CuInS_2_/ZnS	Aspartic acid	7.8 × 10^−8^ M	8.3 × 10^−7^–3.3 × 10^−4^ M	2023	[164]
FL intensity turn-on	CuInS_2_	Zn^2+^	1.99 ppb	0–800 nmol/L	2019	[165]
FL intensity turn-off	AgInS_2_	Gallic acid	20 nM	NA	2019	[166]
FL intensity turn-on	AgInS_2_	Ascorbic acid	45 nM	0–4.2 μM	2019	
FL intensity turn-on	Si QDs–Ag NC	H_2_S	53.6 nM	1.125–17 μM	2023	[167]
FL intensity turn-on	Si QDs–Ag NC	CH_3_SH	56.5 nM	1.125–17 μM	2023	
**Sensing** **mechanism**	**QD type**	**Target analyte**	**FL lifetime**	Δτ **(%)**	**Year**	**Ref.**
Time-gated detection	CuInS_2_	Biological autofluorescence	~100–300 ns	NA	2019	[168]
Time-gated detection	ZnCuInSe/ZnS	Circulating tumor cells	~150–300 ns	NA	2019	[169]
pH-dependent surface state modulation	AgInS_2_/ZnS	Intracellular pH	~284–157 ns	~127 nm (~45%)	2022	[170]
FRET	AgInS_2_/ZnS	Cyanine dyes	~130–80 ns	~38%	2021	[171]
Time-gated detection	Si QDs	Autofluorescence suppression	~10–100 μs	NA	2018	[161]
Time-gated detection	Si QDs	Deep-tissue imaging (NIR-II window)	~5–20 μs	NA	2015	[172]
Optomechanical time-gated detection	Si QDs	Background-free FL imaging	μs-scale	NA	2019	[173]
**Sensing** **mechanism**	**QD type**	**Target analyte**	**D-A pair**	**FRET** **efficiency**	**Year**	**Ref.**
FRET-based sensing	InP/ZnS	Arginine kinase	InP/ZnS QDs (D)-Au NPs (A)	NA	2021	[174]
	InPZnS/ZnSe/ZnS	Prostate-specific antigen (PSA)	Tb-antibody (D)-QD-antibody conjugate (A)	NA	2016	[175]
	AgInS_2_/ZnS	Atenolol	AIS/ZnS QDs (D)-Au NPs (A)	84%	2021	[176]
	AgInS_2_/ZnS	Photosensitizer	AIS/ZnS (D)-AlPc (A)	80%	2022	[160]
	Si QDs (plasmon-enhanced)	Inter-QD energy transfer	Si QDs (D)-Si QDs (A)	~41–46%	2021	[177]

N/A: not available.

## 5. Conclusions and Future Outlook

This review summarizes recent research findings on the evolution of several selected classes of red/NIR emissive, Cd-free QDs from conceptual alternatives into technically mature platforms. They truly possess the potential to replace Cd-based materials in the fields of advanced photonics and nanomedicine. The demand for Cd-free QDs continues to grow in response to increasingly stringent regulations (i.e., RoHS) and the requirement for intrinsic biocompatibility. Specifically, Cd-based QDs can emit toxic ions in cellular environments, even when they are coated with inorganic shells. Consequently, rapid advancements have been made across non-toxic material systems, including exemplary group III-V (InP), group I-III-VI (CuInS_2_, AgInSe_2_, and so on), group-IV (Si, Ge), and even CDs or GQDs. Despite these achievements, each material class still suffers from challenges of scalability and ultimate device performance due to intrinsic physicochemical limitations and the complexity of synthesis (Table 8).

For optoelectronic devices, such as QLEDs, the main challenge is to ensure emission linewidth and operational stability at high current densities. InP QDs, currently the leading Cd-free emitters, generally achieve PLQYs exceeding 90%. However, achieving a narrow FWHM (i.e., less than 40 nm), comparable to CdSe QDs, requires a highly precise core/shell design. The large lattice mismatch between the InP cores and the ZnS shells causes strain-related defects, which necessitate a complex multilayer structure (e.g., ZnSe buffer layers) or advanced core protection strategies. Future development will rely on non-destructive synthesis, including low-temperature InP growth and in situ core stabilization, and a transition from batch synthesis and spin-coating to scalable continuous flow fabrication and patternable deposition compatible with large-area displays [178]. The synthesis of I-III-VI QDs (i.e., CIS and AIS) is relatively straightforward, and high PLQYs can be achieved. However, they are fundamentally limited by defect-mediated emission, which results in broad linewidths (>100 nm), making display color purity requirements incompatible. These materials are more suitable for applications, such as general solid-state lighting, rather than competing with InP in the field of high-end displays. Future research should focus on precise defect engineering through stoichiometric control and management of lattice disorder to narrow FWHM and improve color reproducibility. Meanwhile, CDs or GQDs offer superior sustainability, low toxicity, and synthetic flexibility compared to those of inorganic QDs. However, their solid-state properties are limited by heterogeneous emission mechanisms and aggregation-caused FL quenching (ACQ). To address these issues, advanced surface engineering strategies, including doping of heteroatoms, rigid cross-linking, and emission state separation, are required to enable efficient and stable red/NIR emission in the condensed phase.

In the case of solar cell applications, although not covered in this review, the presence of toxic heavy metals also remains a concern, despite their excellent power conversion efficiencies exceeding >12% [179,180]. In particular, PbS, PbSe, and CdSe QDs exhibit a large Bohr exciton (i.e., 18 nm), high absorption coefficient, and superior charge transport characteristics, enabling them to achieve high efficiencies for solar cells. By contrast, Cd-free alternatives, such as InP [181,182], CIS [183,184], and AIS [185,186], QDs have been explored, but their device performance is yet limited by relatively low absorption coefficients, challenges in controlling surface defects, and inefficient charge transport properties. Moreover, the best performance is typically achieved when the absorption spectral range is limited to the visible range and not the NIR. Stability to oxygen/moisture should also be addressed compared to conventional Pb-based QDs. Above all, despite significant progress, a substantial technical gap exists between the thin-film device and the QD-based device, regardless of constituent materials, indicating that further research and development are required for QDs. Several good review papers well establish materials design and the required characteristics for QD solar cells [187,188,189].

Group-IV QDs (Si and Ge) occupy a distinct and strategically important niche in silicon photonics. Their intrinsic biocompatibility and full compatibility with complementary metal–oxide–semiconductor (CMOS) processing enable monolithic integration of on-chip light sources and detectors operating in the NIR-II windows (~1.3–1.7 μm). Future advances will rely on site-controlled epitaxial growth, interface perfection, and exploitation of type-II band alignment (e.g., Ge–core/Si–shell architectures) to enhance radiative efficiency for high-speed optical interconnects and IR sensing technologies [190]. Meanwhile, the indirect bandgap properties of group-IV materials represent another critical challenge in the application of LEDs and semiconductor lasers [191,192]. Unlike direct bandgap materials, such as InP, the valence band maximum and conduction band minimum in group-IV materials occur at different momentum values in *k*-space rather than at the same point, leading to intrinsically slow radiative recombination. For example, in Auger recombination, the energy released by an electron–hole pair (EHP) can be transferred to a third carrier (an electron or hole) as kinetic energy, which subsequently dissipates as heat [193]. Shockley–Read–Hall (SRH) recombination [194] can also occur, where electrons and holes recombine through defect states within the crystal lattice, again losing energy by non-radiative recombination. Overcoming these limitations might involve strategies such as enhanced quantum confinement [195] through nanostructuring, strain engineering [196] to modify band structure, integrating hexagonal phases, or alloying [197] to partially relax momentum mismatch and improve radiative recombination efficiencies.

In biological applications, the red/NIR emissions offer deep-tissue penetration and reduced background autofluorescence; however, long-term surface stability under various physiological conditions remains a major challenge [198]. While group-IV (Si, Ge) QDs, GQDs, and CDs exhibit excellent intrinsic biocompatibility, the InP and AgInS_2_ QDs can still induce cytotoxicity if surface passivation is incomplete or surface ligands are inactive [199]. Moreover, most QDs synthesized by the hot-injection method require post-synthetic ligand exchange to achieve aqueous dispersibility, which often leads to degradation in optical stability. Future research must prioritize the development of robust, single-step aqueous solutions or photochemical synthesis capable of withstanding complex biological environments and obtaining stable water-dispersible QDs without extensive post-processing. Beyond imaging, the future of red/NIR-emissive QDs in the biomedical field is becoming increasingly multimodal. The potent NIR absorption properties of CDs and Ge QDs enable image-guided photothermal therapy. Integrating them with targeting ligands, peptides, or aptamers provides effective ways to simultaneously sense, track, and treat. To achieve truly quantitative biodistribution and reliable in vivo detection, precise control over pharmacokinetics, elimination pathways, and long-term stability is required, which could likely be achieved through advanced liposomal or polymeric encapsulation strategies [200].

Note that within the very broad material category, several Cd-free (and even lead (Pb)-free, within the broader category) material systems have been selectively discussed due to their relatively superior performance and research maturity. Other emerging classes of inorganic nanocrystals, such as Zn-based chalcogenides (e.g., ZnSe, ZnTe) and pnictogen-based QDs (e.g., Zn_3_P_2_, CuSb_2_S_2_), also serve as promising alternatives for their applications in the red/NIR regions. Although ZnSe-based QDs offer low toxicity, their inherently wide bandgaps often result in lower efficiency in red/NIR emissions. For pnictogen-based QDs, chemical stability under ambient conditions should be addressed. The Pb-free perovskite QDs, particularly tin (Sn)-based systems (e.g., CsSnX_3_), are another good candidate due to the smaller ionic radius, distinct electronegativity of Sn^2+^ compared to that of Pb^2+^, and intrinsically narrow bandgaps [201,202]. One of the challenges that has to be addressed is the rapid oxidation of Sn^2+^ to Sn^4+^, which introduces p-type defects and non-radiative recombination centers [203]. For readers interested in a more specialized discussion of these emerging inorganic QD families, several comprehensive reviews provide excellent summaries of their synthesis, photophysical properties, and applications [204,205].

In addition, it is important to note that “Cd-free” does not directly imply “risk-free” or “non-toxic”. For example, the InP QDs contain In, a transition metal that might exhibit pulmonary and systemic toxicities, depending on its chemical form. Leaching of constituent ions, such as selenium (Se) or sulfur (S), may also occur through oxidative dissolution of the shell (e.g., ZnS or ZnSe) under acidic microenvironments. The leaching rate can be accelerated by the degradation of organic ligands, such as oleic acid and thiols, by endogenous proteins (e.g., albumin) in the bloodstream. In addition to elemental composition, the size and surface properties of QDs can also influence the toxicity. The QDs smaller than 10 nm might penetrate the cellular membrane. Depending on surface charge (both negative and positive), the QDs non-specifically interact with cytoplasmic proteins, which leads to protein misfolding or enzymatic inhibition. Reactive oxygen species (e.g., ·OH) generated through the photooxidation of QDs can cause oxidative stress, lipid peroxidation, and subsequent DNA damage. Therefore, the term “risk-free” or “non-toxic” should be understood within a framework of a comprehensive human hazard assessment. First, in vivo testing protocols using standardized animal models can be utilized to evaluate long-term physiological changes, such as weight fluctuations or organ-specific accumulation over an extended period. Second, molecular-level toxicology analysis, such as RNA sequencing and proteomics, should be carried out to understand the underlying mechanism of potential toxicity. Third, the claim of “low-toxicity” should be validated through standardized protocols in compliance with ISO 10993 (biological evaluation of medical devices) [206] to ensure that the materials remain stable and non-hazardous throughout their operational lifespan.

Ultimately, the core objective of all Cd-free QD systems is to achieve performance equivalent to or better than Cd-based QD systems without compromising environmental and biological safety. Realizing this goal requires a more rigorous, data-driven approach to materials design, utilizing computational modeling and machine learning to elucidate correlations between subtle compositional and surface state variations. Such strategies are essential for accelerating the discovery and optimization of robust, red/NIR-emissive, and environmentally compliant nanomaterials for next-generation photonics and biomedical technologies.

## Figures and Tables

**Table 1:** Properties of red-emissive, graphene- or carbon-based QDs.

Method	Precursor	Size [nm]	PL peak [nm]	FWHM [nm]	PLQY [%]	Year	Ref.
Low temperature pyrolysis	VCX-72 carbon black, nitric acid, PEI_600_	57.31 ± 8.90	622	100	9.7	2017	[95]
Amidative cutting	Graphite, nitric acid, 4-(methylthio)aniline	2.7	620	50	21	2016	[60]
Solvothermal	1,3,6-trinitropyrene, boric acid	4.0	617	42.5	17.1	2021	[59]
Solvothermal	*p*-phenylenediamine	10.0	625	58	16.3	2015	[25]
Solvothermal	*p*-phenylenediamine, urea	2.6	625	68	24	2016	[26]
Solvothermal	AEAPMS ^1^, citric acid anhydrous	3	648	83	9.6	2017	[27]
Electrolysis	Graphite, K_2_S_2_O_8_	3	610	72	11.2	2015	[96]
Hydrothermal	*p*-phenylenediamine, melamine	3.9 ± 0.6	624	54	30	2021	[92]
Solvothermal	*o*-phenylenediamine, L-cystine	2.97	648	58	35.7	2019	[61]
Solvothermal	Pulp-free lemon juice	4.6	631	N/A	28	2017	[62]
Solvothermal	Taxus leaves	2.7 ± 0.7	673	20	31	2020	[67]
Solvothermal	Critic acid	3.26	667	N/A	60.73	2022	[63]
Solvothermal	*o*-phenylenediamine	2	620	24	14	2021	[64]
Solvothermal	Mulberry leaves	3	676	20	73	2022	[65]
Solvothermal	Thiourea, critic acid	4–7	610	N/A	24	2020	[66]
One-pot polymerization	*p*-phenylenediamine	1.2–2.8	600	N/A	7	2018	[94]
Solvothermal	Nitrated pyrenes, boric acid	2.88	595	N/A	65	2023	[97]
Solvothermal	Citric acid, thiourea, ammonium fluoride	3.2	670	N/A	40	2026	[98]
Solvothermal	Critic acid, formamide	2.33	640	N/A	19	2023	[99]
Solvothermal	2, 5-diaminotoluene sulphate	6.7 ± 1.5	611	N/A	24.1	2026	[100]
Solvothermal	2,3-diaminobenzoic acid	4.63	599	N/A	33.7	2024	[101]
Ultrasonication, ball milling	Graphite	41	610	N/A	N/A	2024	[102]

N/A: not available.

**Table 3:** Properties of red/NIR-emissive, Cu-In core-based QDs.

Composition	Method	Precursor	Size [nm]	PL Peak [nm]	FWHM [nm]	PLQY [%]	Year	Ref.
CuInS_2_/ZnS	Hot-injection	CuI, In(OAc)_3_, DDT, Zn(OAc)_2_	4.6	618–814	Broad	74–82	2023	[17]
CuInSe_2_/ZnS/ZnS	Hot-injection	CuI, In(OAc)_3_, Zn(OAc)_2_, Se pellets	8.8	705–728	96–114	80	2022	[18]
CuInS_2_/ZnS	Hot-injection	CuI, In(OAc)_3_, DDT, S (elemental) + OLA, Zn(OAc)_2_	6.9	868	Broad	91	2019	[113]
CuInSe_2_/ZnS	Hot-injection	CuI, In(OAc)_3_, Se (dissolved in OAm and DDT), Zn(OAc)_2_, DDT	5.4–10.3	920–1224	Broad	21.8	2020	[117]
CuInS_2_/ZnS	Aqueous co-precipitation	CuCl_2_·2H_2_O, InCl_3_·4H_2_O, Na_2_S, Zn(OAc)_2_·2H_2_O, MPA	3.18–4.57	640	98	22.9	2021	[115]
N-doped GQDs/CuInS_2_/ZnS	Aqueous co-precipitation	CuCl_2_·2H_2_O, InCl_3_·4H_2_O, Na_2_S, Zn(OAc)_2_·2H_2_O, MPA	4.5	643	98	38	2021	
CuInGaS_2_	Hot-injection, cation exchange	CuSO_4_·5H_2_O, DDT, InCl_3_, GaCl_3_, TOP, DPP, TPP	5.5	~1150	N/A	N/A	2019	[120]
CuInS_2_	Hot-injection	CuI, In(Ac)_3_, DDT	3.4	640	98	0.7	2018	[116]
CuInS_2_/ZnS	Hot-injection	CuI, In(OAc)_3_, DDT, Zn(stearate)_2_, ODE	2–4	680–780	110	67	2011	[118]
CuInS_2_/ZnS	Hot-injection	CuI, In(OAc)_3_, DDT, Zn(OAc)_2_ (Zn-oleate), OLAm, OA, ODE (stoichiometric) Cu(OAc), In(St)_3_, S (ODE), DDT, OA, ODE (Cu-deficient)	NA	600–800	Broad	55	2017	[41]
CuInS_2_	Aqueous co-precipitation	Cu(CH_3_COO)_2_, InCl_3_, Na_2_S·9H_2_O, MPA, NaOH	3–4	676	115	N/A	2023	[121]
CuInS_2_/ZnS	Hot-injection	CuI, In(OAc)_3_, DDT, Zn(St)_2_	4.0 ± 0.2	550	125	81	2015	[122]
CuInS_2_	Hot-injection	CuI, In(OAc)_3_, DDT	4.0 ± 0.2	623	125	23	2015	
CuInS_2_	Hot-injection	CuI, In(OAc)_3_, DDT	2.3	730	300	75–95	2023	[114]
CuInS_2_/ZnS	Hot-injection, cation exchange	Cu(OAc), DDT, InCl_3_/In(NO_3_)_3_·H_2_O/In(OAc)_3_/In(acac)_3_, Zn(St)_2_, TOP, TOPO, elemental S (in TOP), ODE	4.5–8.1	750–1100	200–300	75 (at 820 nm)	2017	[119]
CuInS_2_/ZnS	Hot-injection, cation exchange	Cu(OAc), DDT, InCl_3_/In(NO_3_)_3_·H_2_O/In(OAc)_3_/In(acac)_3_, Zn(St)_2_, TOP, TOPO, elemental S (in TOP), ODE	4.5–8.1	750–1100	200–300	25 (at 1050 nm)	2017	

N/A: not available.

**Table 4:** Properties of red/NIR-emissive, Ag-In core-based QDs.

Composition	Method	Precursor	Size [nm]	PL Peak [nm]	FWHM [nm]	PLQY [%]	Year	Ref.
AgIn_3_Se_5_/ZnSe//ZnS	Hot-injection	AgNO_3_, In(OAc)_3_, Se, Zn(OAc)_2_, S	3–3.8	670	148	~46	2024	[129]
AgIn_x_Ga_1-x_S_2_	Hot-injection	Ag(OAc), In(OAc)_3_, Ga(DDTC)_3_	3.5–3.9	112–127	588–601	9–22	2023	[130]
AgIn_x_Ga_1-x_S_2_	Hot-injection	Ag(OAc), InCl_3_, Ga(DDTC)_3_	2.9–4.4	37–155	534–655	25–47	2023	
AgIn_5_S_5_/ZnS	Aqueous synthesis	AgNO_3_, InCl_3_, Na_2_S, Zn(OAc)_2_, MPA	3.1–4.5	~130	635	~30	2024	[123]
AgIn_5_S_8_	Hot-injection	AgNO_3_, In(acac)_3_, S	~2	N/A	660	N/A	2023	[131]
AgIn_5_S_8_/ZnS	Hot-injection	AgNO_3_, In(acac)_3_, S, ZnO	3–4	N/A	560	54.4	2023	
AgInS (Ag/In ratio of 1/5)	Hot-injection	AgNO_3_, In(NO_3_)_3_·*x*H_2_O, Na_2_S·9H_2_O	2.1	110	550–640	3.8–33.5	2020	[132]
AgInS/ZnS (Ag/In ratio of 1/5)	Hot-injection	In(NO_3_)_3_·*x*H_2_O, AgNO_3_, Na_2_S·9H_2_O, Zn(OAc)_2_·2H_2_O	2–4	103, 120	540–610	65–77.8	2020	
AgIn_4_S_10_/2MPA	Aqueous synthesis	AgNO_3_, In(NO_3_)_3_·*x*H_2_O, Na_2_S, 2MPA	5.11–6.59	79–219	630–660	16.7–22.1	2022	[133]
AgIn_10_S_10_-PEI/2MPA	Aqueous synthesis	AgNO_3_, In(NO_3_)_3_·*x*H_2_O, Na_2_S, 2MPA, PEI	4.4–7.8	147	617	18.9–21.7	2022	
AgInGaSSe	Aqueous synthesis	Ag(OAc), In(acac)_3_, Ga(acac)_3_, thiourea, selenourea	3.0–3.8	N/A	651–856	25	2021	[125]
AgInGaSSe/Ga_x_	Aqueous synthesis	Ag(OAc), In(acac)_3_, Ga(acac)_3_, thiourea, selenourea	3.5–4.2	N/A	580–790	50	2021	
PVP-capped AgInS_2_	Solvothermal	AgNO_3_, InCl_3_, Na_2_S·9H_2_O	2	100–120	575–597	N/A	2022	[134]
Ag_0.34_Zn_0.59_InS_2_/ZnS_1.69_	Hot-injection	AgNO_3_InCl_3_, Na_2_S·9H_2_O, Zn(CH_3_COO)_2_	4.8 ± 1	136	696	55	2020	[135]
TOP-treated AIGSe@GaS_x_ (*x* = 0.67/*y* = 0.75)	Solvothermal	Ag(OAc), In(acac)_3_, In(OAC)_3_, Ga(acac)_3_, (H_2_N)_2_CSe, (H_2_N)_2_CS	4.3–5	0.14–0.21 (eV)	800	38	2020	[136]
Mn:AIGZS Mn (2.5%)	Solvothermal	AgNO_3_, In(NO)_3_·*x*H_2_O, Ga(NO_3_)_3_·*x*H_2_O, Zn(DDTC)_2_,MnCl_2_·4H_2_O	1.9 ± 0.5	N/A	612	41.3	2021	[128]
AgIn_5_S_8_/ZnS	Aqueous synthesis	AgNO_3_, In(acac)_3_, Zn stearate, S	3.0, 3.3	91	585, 670	31, 35	2017	[137]
AgIn_x_Ga_1-x_S_2_	Hot-injection	Ag(OAc), InCl_3_, In(DDTC)_3_, Ga(DDTC)_3_	4.9	151	669	37	2024	[138]
AgInSe/ZnS	Aqueous synthesis	AgNO_3_, InCl_3_, Na_2_SeSO_3_, Na_2_SO_3_	2.2 ± 0.1	120	650	12	2020	[139]
Zn_0.30_Ag_1_In_1.39_ Ga_0.32_/InS_0.15_ (core/shell I)	Aqueous synthesis	AgI, InI_3_, ZnI_2_, Ga(acac)_3_, OAm	5.27	78	628	86.2	2023	[124]
AgCuInS/GaS	Hot injection	Ag(OAc), In(DDTC)_3_, InCl_3_, GA(DDTC)_3_	8.4	60	650	65	2023	[127]
Ag(InxGa_1−x_)S_2_/GaS_y_	Aqueous synthesis	Ag(OAc), In(OAc)_3_, Ga(DDTC)_3_	4.5–11	32–42	498–601	28–59	2021	[126]

N/A: not available.

**Table 5:** Properties of red/NIR-emissive, silicon, or germanium QDs.

Composition	Method	Precursor	Size [nm]	PL Peak [nm]	FWHM [nm]	PLQY [%]	Year	Ref.
Si	Template	Dodecyl	2.9–3.6	645–712	N/A	48	2018	[21]
Si	Zintl salt oxidation	n-butyl	2.2	650	N/A	50	2018	[22]
Si	Electrochemical etching	Complex shell	5–8	621	N/A	55	2016	[75]
Si	Plasma	Dodecyl	4.5	850	N/A	67	2020	[76]
Si	Non-thermal plasma	Si/SiO_2_	4	825	N/A	90	2019	[77]
Si	HSQ	Decyl, Cl	3.2	680	N/A	54	2022	[78]
Si	HSQ	Dodecyl	4.2	867	N/A	37	2017	[79]
Si	HSQ	Dodecyl	2.9	743	N/A	15	2017	
Si	HSQ	Decyl	2.4	740	N/A	77	2023	[80]
Ge	MBE	Ge solid source	<4	1560	70	N/A	2016	[23]
Ge	MBE	Ge solid source	<4	1488	70	N/A	2016	
Ge	MBE	Ge solid source	<4	1352	70	N/A	2016	
Ge	MBE	Ge solid source	<4	1323	70	N/A	2016	
Ge	Colloidal	GeI_2_ + OAm/ODE/TOP	3–18	930–1550	N/A	N/A	2025	[24]
Ge	RPCVD	SiH_4_/GeH_4_	18 ± 3	1500–1870	84	N/A	2023	[140]

N/A: not available.

**Table 6:** Summary of FL sensors using CDs or GQDs.

**Sensing** **mechanism**	**QD type**	**Target analyte**	**LOD**	**Linear range**	**Year**	**Ref.**
FL intensity turn-off	B, N, S-co-doped CDs	Fe^3+^	90 nM	0.3–546 μM	2017	[152]
FL intensity turn-on	Hg^2+^–GQDs	CN^−^	3.10 μM	5–15 μM	2021	[154]
FL intensity turn-off	Hg^2+^–GQDs	[Fe(CN)_6_]^3−^	9.48 μM		2021	
**Sensing** **mechanism**	**QD type**	**Target analyte**	**D-A pair**	**FRET** **efficiency**	**Year**	**Ref.**
FRET-based sensing	CDs	17β-estradiol	CDs (D)-Au NPs (A)	NA	2023	[153]
	CDs	Biomolecule (biosensing target)	CDs (D)-Au NPs (A)	NA	2023	
	GQDs-SiO_2_ NP	Energy transfer system (optoelectronic)	SiO_2_ NP (D)-GQDs (A)	~78%	2016	[155]

**Table 8:** Performance metrics and development outlook by QD class.

QD Class	Dominant PL Mechanism	Key Performance	Technical Challenge	Future Opportunity
Graphene or carbon-based	Localized sp^2^ carbon subdomain/surface states	Excellent aqueous stability, ultra-low toxicity,	Aggregation-caused quenching (ACQ) in solid state	Solid-state lighting (WLEDs with high CRI) via surface engineering, multi-modal nanomedicine/biosensing
Indium phosphide (InP)	Excitonic (quantum confinement)	High PLQY (>90%), narrow FWHM (<40 nm)	Core dissolution/defect formation during high-temperature shell growth	Industrial scale-up, next-generation high color purity displays
Copper/silver–indium (Cu-In/Ag-In)	Defect-mediated (donor–acceptor pair recombination)	High PLQY (up to 95% for CuInS_2_), tunable NIR emission	Broad FWHM (>100 nm), defect control	NIR-II bioimaging/sensors through precise defect engineering
Silicon (Si)	Quantum confinement/ surface states	High PLQY (up to 90%), ultra-low cytotoxicity	Indirect bandgap, surface oxidation instability	CMOS-compatible devices, cost-effective, large-scale manufacturing
Germanium (Ge)	Quantum confinement	Intrinsic NIR-II emission (1310–1770 nm), high PTT efficiency	Integration stability with Si substrate, lower PLQY	On-chip integrated photonics, image-guided PTT

## Data Availability

No new data were created or analyzed in this study.
